# Policosanol alleviates chronic stress-induced growth impairment via gut microbiota-metabolite interactions: insights from 16S rRNA sequencing and LC-MS metabolomics

**DOI:** 10.3389/fnut.2025.1685003

**Published:** 2026-01-05

**Authors:** Ruxia Wang, Linfang Hu, Yue Tu, Zhenya Zhai, Kaimin Niu, Xiongchang Guo, Lichuang Cai, Jianping Liu

**Affiliations:** Institute of Biological Resource, Jiangxi Academy of Sciences, Nanchang, China

**Keywords:** policosanol, stress, gut microbiome, metabolites, growth performance

## Abstract

**Background/objectives:**

Policosanol, a bioactive compound derived from rice bran wax, has demonstrated potential for alleviating stress, yet its underlying mechanisms remain elusive. This study aimed to elucidate its role in mitigating chronic stress-induced growth impairment and to explore its interactions with the gut microbiota and metabolomics.

**Methods:**

Male rats were subjected to a 4-week chronic restraint stress protocol with or without policosanol supplementation (2 mg/kg/day). Systemic responses were evaluated by measuring growth parameters (including weight gain and muscle mass), serum biomarkers [cortisol and catecholamines (CA)], 16S rRNA sequencing (for cecal microbiota analysis), and LC-MS metabolomics (for cecal metabolite profiling).

**Results:**

Stress induced a significant reduction in weight gain (−11.0%, *p* < 0.05) and a marked elevation of serum cortisol (+86.2%) and CA (+88.3%, both *p* < 0.05). Policosanol treatment restored weight gain to 85.5% of control levels (*p* < 0.05) and reduced cortisol and catecholamine levels by 29.5% and 26.8%, respectively (both *p* < 0.05). Stress-induced alterations in gut microbiota included a 4.1-fold increase in p_Verrucomicrobiota and a 3.8-fold increase in *g_Akkermansia*, along with metabolite changes such as a 4.2-fold elevation in Proscillaridin and a 65% decrease in Phenylacetylglutamine (PAGln) (both *p* < 0.05). Policosanol supplementation normalized gut microbiota composition (p_Verrucomicrobiota decreased by 36%, *p* < 0.05) and restored metabolite levels (PAGln increased by 80%, *p* < 0.01). Negative correlations were observed between *g_Akkermansia* abundance and weight gain (*p* < 0.01), while PAGln positively correlated with growth (*p* < 0.05) and negatively correlated with GSH-Px (*p* < 0.001), cortisol (*p* < 0.001), and CA (*p* < 0.001). Moreover, the *g_Bacteroides*–PAGln axis exhibited a strong interaction (*p* < 0.001).

**Conclusion:**

Policosanol mitigates stress-induced growth impairment by modulating gut microbiota (e.g., reducing p_Verrucomicrobiota and *g_Akkermansia* abundances) and restoring metabolite levels (e.g., increasing PAGln). The coregulation of the gut microbiota and metabolome was highlighted by a strong correlation between *g_Bacteroides* and Phenylacetylglutamine (PAGln), suggesting a potential functional interaction that may contribute to the anti-stress effects of policosanol, though causality remains to be established.

## 1 Introduction

Rice, one of the most widely cultivated crops globally, accounts for approximately one-third of the world’s grain production. In 2021, China, the world’s largest rice producer, generated an impressive 148.3 million metric tons of refined rice, according to the 2022 agricultural production data published by China’s Ministry of Agriculture. Rice bran, constituting 6%–10% of the entire rice kernel, is a rich source of nutrients, including oils, proteins, dietary fibers, and bioactive compounds such as phytosterols, sterols, and policosanol. These components confer numerous health benefits to rice bran, ranging from cholesterol reduction to cardiovascular protection (1–3). However, in China, the use of rice bran remains predominantly limited to livestock feed, with an alarmingly low effective utilization rate of less than 20%. This underutilization highlights the urgent need to explore the active constituents of rice bran to unlock its full potential and thereby enhance its utilization efficiency.

Policosanol, also known as octacosanol or high-molecular-weight alcohols, is a prominent active ingredient in rice bran. It exhibits a wide array of beneficial properties, such as lipid-lowering, anti-inflammatory, and antioxidant activities, and finds extensive applications in food, pharmaceutical, and cosmetic industries (4–6). Consequently, the biological functions and development potential of policosanol have become focal points of contemporary scientific research. Previous studies have demonstrated that policosanol can improve serum lipid profiles by reducing total cholesterol and low-density lipoprotein cholesterol levels, and can inhibit weight gain and fat accumulation induced by a high-fat diet, thus offering potential therapeutic benefits against hyperlipidemia and cardiovascular diseases (5, 7). Moreover, policosanol derived from rice bran wax has shown potent anti-inflammatory effects by mitigating the risk of atherosclerosis through inhibition of platelet aggregation and modulation of gut microbiota composition, alleviating symptoms associated with inflammatory bowel disease (4, 8). Regarding its anti-stress properties, policosanol, owing to its long-chain fatty alcohol structure, can effectively integrate into the lipid bilayer of cell membranes, enhancing membrane stability and reducing damage caused by free radicals (9). For instance, policosanol can inhibit the generation of reactive oxygen species in 3T3-L1 adipocytes, protecting cells from oxidative stress; simultaneously, by regulating intracellular antioxidant enzyme activities, such as superoxide dismutase (SOD) and glutathione peroxidase (GSH-Px), it further strengthens the cellular antioxidant defense system (10). Studies have also indicated that policosanol can ameliorate alcohol-induced liver damage by enhancing antioxidant functions and attenuating oxidative stress reactions caused by alcohol consumption (11, 12).

While conventional antioxidants, such as vitamin E and ascorbic acid, have been extensively studied for their efficacy in alleviating oxidative stress through direct free-radical scavenging, their mechanisms are often singular and limited in addressing the multifactorial nature of chronic stress. In contrast, policosanol, a natural mixture of long-chain alcohols derived from rice bran wax, exhibits multi-mechanistic anti-stress effects beyond conventional antioxidant activity. Its unique chemical structure enables its incorporation into cell membranes, enhancing lipid bilayer stability and reducing susceptibility to oxidative damage (4, 9). Moreover, unlike many plant-derived bioactive compounds such as resveratrol or curcumin, policosanol demonstrates an exceptional safety profile and favorable tolerability, making it particularly suitable for long-term use in stress mitigation protocols. Recent evidence further underscores its ability to modulate gut microbiota composition (7, 8), representing a novel pathway rarely reported for traditional anti-stress agents. This microbiota-targeting effect, combined with its systemic regulation of neuroendocrine and redox pathways (5, 6), positions policosanol as a holistic therapeutic candidate capable of concurrently addressing physiological, metabolic, and microbial dimensions of chronic stress. Nevertheless, current research on the anti-stress effects of policosanol has primarily focused on its antioxidative capacity, and the comprehensive mechanisms underlying its actions remain incompletely understood.

This study aimed to investigate the ameliorative effects of policosanol on cellular oxidative stress and physical stress responses induced by restraint in rats. In our previous investigations, we observed that dietary supplementation with policosanol modulated gut microbiota composition (7), which in turn could influence the body’s stress responses either directly through microbiota alterations or indirectly via microbial metabolites (8). Therefore, we employed gut microbiome analysis and metabolomics techniques to elucidate the mechanisms by which policosanol enhances the organism’s ability to cope with stress, shedding light on the potential molecular pathways involved in growth and development. By delving deeper into the mechanistic actions of policosanol against stress, our objective was to provide valuable insights for advancing targeted therapeutic strategies in this field.

## 2 Materials and methods

### 2.1 Materials and chemicals

**Figure 1:**
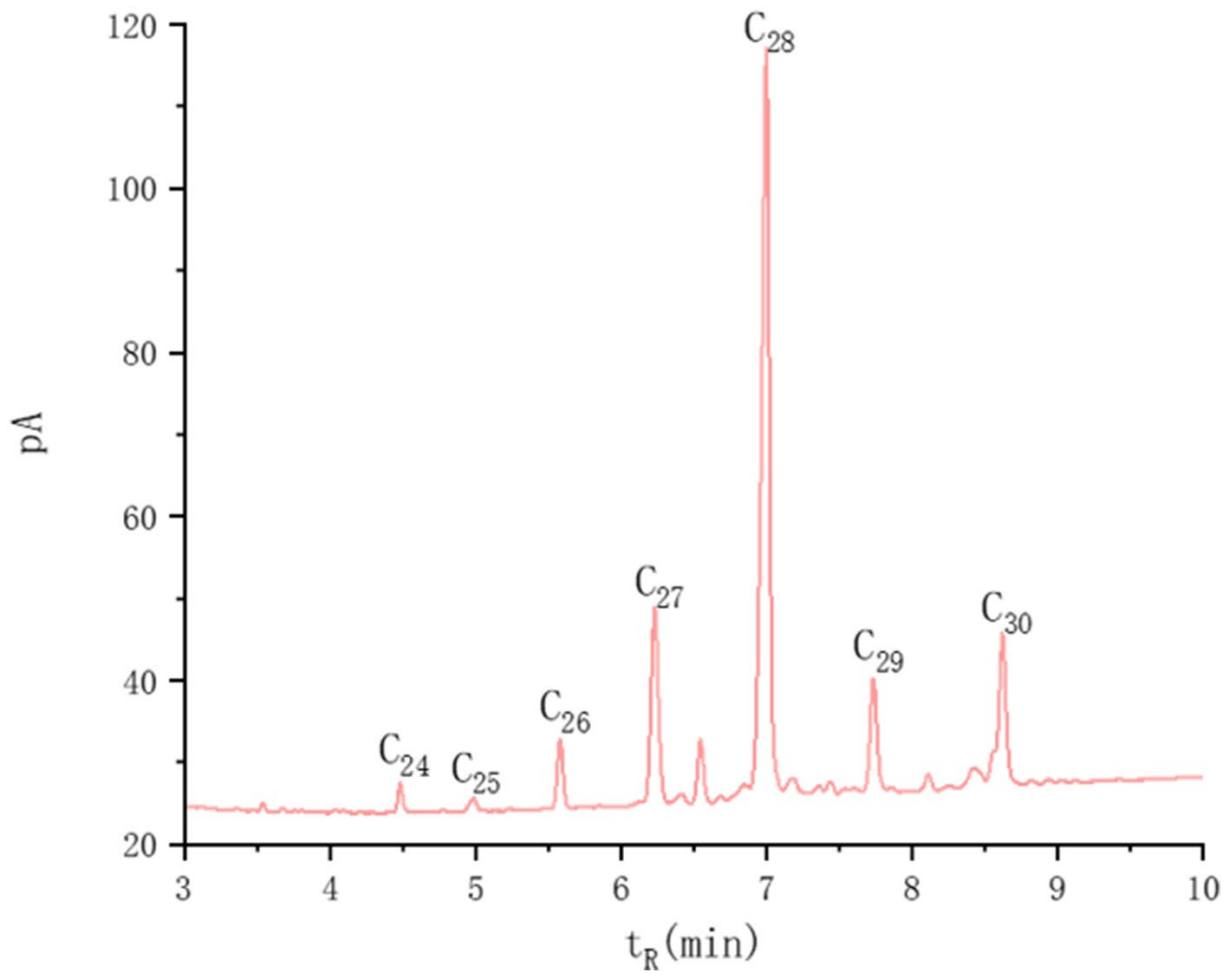
Chromatographic profile and compositional analysis of policosanol derived from rice bran wax. Qualitative analysis of the main components in policosanol samples was conducted using high-grade alcohols as reference standards. The mass percentage content of each peak was calculated utilizing the normalization method. The policosanol mixture used in this study had a total content of long-chain alcohols (C22–C30) of >84% (by mass), with octacosanol (C28) as the predominant constituent.

Policosanol was isolated from rice bran wax and analyzed in our laboratory (Figure 1). The policosanol used in this study was composed of docosanol (C22H45OH, 0.16%), tricosanol (C23H47OH, 0.13%) tetradecanol (C24H49OH, 1.21%), pentacosanol (C25H51OH, 0.84%), hexacosanol (C26H53OH, 3.38%), heptacosanol (C27H55OH, 12.43%), octacosanol (C28H57OH, 48.91%), nonacosanol (C29H59OH, 6.51%), and triacontanol (C30H61OH, 10.50%). Rat feed was procured from Trophic Animal Feed High-Tech Co., Ltd. (Nantong, China). Serum and tissue biochemical assay kits for determining the levels of SOD, malondialdehyde (MDA), and GSH-Px in serum and liver tissues were supplied by Suzhou Comin Biotechnology Co., Ltd. (Suzhou, China). ELISA kits for serum cortisol and catecholamines (CA) were provided by Nanjing Boyan Biotechnology Co., Ltd. (Nanjing, China).

### 2.2 *In vitro* experimental design and sample collection

NCTC1469 cells (Procell Life Science and Technology Co., Ltd., Wuhan, China) were cultured at 37 °C and 5% CO_2_ in medium supplemented with 10% fetal bovine serum and 5% penicillin/streptomycin. Subsequently, well-grown NCTC1469 cells were randomly allocated into three groups (*n* = 6 per group): control (Control), stress (Dexamethasone, DEX), and policosanol (DEX + Policosanol). The DEX treatment group received 10 μM dexamethasone, while the DEX + Policosanol group was administered 10 μM dexamethasone and 100 mg/L emulsified policosanol. The Control and DEX groups received an equivalent volume of blank emulsifier. Dosages for dexamethasone and policosanol supplementation were based on preliminary pilot studies. After 24 h of treatment, cell samples were collected, and relevant indicators were measured.

### 2.3 *In vivo* experimental design and sample collection

Male rats (12 weeks old; Hunan Slek Jinda Experimental Animal Co., Ltd., Changsha, China) were housed under specific-pathogen-free (SPF) conditions. All animal procedures were approved by the Animal Ethics Committee of the Jiangxi Academy of Science (Approval No. JXAS-2024-022). Following a 1-week acclimatization period, rats were randomly divided into three groups (*n* = 7 per group): control group (normal diet), stress group, and stress + policosanol group. The randomization was performed using a computer-generated random number sequence. To minimize bias, investigators responsible for the subsequent procedures, data collection, and outcome assessment were blinded to the group allocations throughout the experiment. Detailed compositions of experimental diets are presented in Table 1. All rats had free access to food and water. The chronic stress model was established by daily capture and gastric gavage. The procedure was performed daily between 8:00 and 9:00 a.m. for 4 weeks. To isolate the effect of the stress procedure from the policosanol treatment, the handling was standardized as follows: the control group was left undisturbed in their home cages; the stress group received gastric gavage of the blank emulsifier vehicle; the stress + policosanol group received gastric gavage of emulsified policosanol (2 mg/kg body weight). A detailed schematic of the experimental timeline is provided in Supplementary Figure S2. Rats in the policosanol group received 2 mg/kg body weight of policosanol via gastric gavage daily at 8:00 a.m. Dose justification was based on prior studies (7). The experimental duration was 4 weeks. At the conclusion of the experiment, all rats were anesthetized with isoflurane after a 12-h fasting period. Euthanasia was performed using carbon dioxide (CO₂) asphyxiation in a dedicated chamber. The CO₂ flow rate was carefully controlled to displace 30% of the chamber volume per minute, ensuring a gradual loss of consciousness and minimizing distress. Death was confirmed by the absence of spontaneous breathing, followed by cervical dislocation as a secondary physical method of euthanasia. Throughout the experimental period, including the euthanasia procedure, rats were maintained under specific-pathogen-free (SPF) conditions. The housing environment was strictly controlled at a temperature of 22 °C–25 °C and a relative humidity of 50% ± 10%, with a 12-h light/dark cycle. Blood samples were collected from the venous plexus of the eye and centrifuged at 1,000 × *g* for 10 min at 4 °C to obtain serum. Concurrently, the animals were sacrificed, and thigh muscles were harvested and weighed. Samples of appendix contents and liver tissues were rapidly frozen in liquid nitrogen and stored at −80 °C until analysis.

**Table 1:** Feed ingredients and nutritional levels.

Feed ingredients	Content
Casein	200
L-Cystine	3
Starch, corn	350
Fructose, monohydrate	169
Sucrose	100
Lodex10	85
Cellulose	50
Soybean oil	25
Lard	20
Palm oil	0
Mineral mix S10026B	50
Vitamin mix, V10001C	1
Choline bitartrate	2
Cholesterol	0
Total	1,055

Nutritional levels	gm%	Kcal%
Protein	19.24	20.11
Carbohydrate	66.82	69.85
Fat	4.27	10.03
Total	/	100
Kcal/gm	4.49	

### 2.4 Cell viability assay

NCTC1469 cell viability was assessed using the Cell Counting Kit-8 (CCK-8) according to the manufacturer’s instructions (*n* = 6 per group).

### 2.5 Biochemical analyses

Serum concentrations and hepatic levels of SOD, MDA, and GSH-Px were determined using commercial biochemical assay kits according to the manufacturers’ guidelines (*n* = 7 per group). Serum levels of cortisol and CA were quantified following the provided instructions (*n* = 7 per group).

### 2.6 16S rRNA analysis of gut microbiota

Wet-lab procedures: DNA extraction was performed using the OMEGA Soil DNA Kit (D5635-02, USA). Extracted DNA was evaluated for molecular size by 0.8% agarose gel electrophoresis and quantified using a NanoDrop spectrophotometer. For bacterial community profiling, the highly variable V3–V4 region (~480 bp) of the bacterial 16S rRNA gene was selected for sequencing. PCR amplification utilized specific primers targeting the bacterial 16S rRNA V3–V4 region: 338F (5′-barcode + ACTCCTACGGGAGGCAGCA-3′) and 806R (5′-GGACTACHVGGGTWTCTAAT-3′). PCR reactions were conducted using NEB Q5 high-fidelity DNA polymerase, with cycling parameters as follows: pre-denaturation at 98 °C for 5 min, followed by 25 cycles (98 °C for 30 s, 53 °C for 30 s, 72 °C for 45 s), and a final extension at 72 °C for 5 min. Amplified products were separated by 2% agarose gel electrophoresis, excised to isolate target fragments, and recovered using the Axygen Gel Recovery Kit. Qualified libraries underwent paired-end sequencing (2 × 250 bp) on the Illumina NovaSeq platform using the NovaSeq 6000 SP Reagent Kit (500 cycles).

#### 2.6.1 Bioinformatic processing

Raw sequencing data were demultiplexed and quality-filtered using the QIIME2 pipeline (version 2023.5). Primer sequences were trimmed using the Cutadapt plugin. Denoising, chimera removal, and generation of amplicon sequence variants (ASVs) were performed using the DADA2 plugin, which models and corrects Illumina sequencing errors to resolve exact biological sequences. This process resulted in a feature table of ASVs and their representative sequences. The average sequencing depth was 65,974 reads per sample (total reads: 1,187,528 across all 18 samples). After quality filtering and denoising, a total number of 3,102 ASVs high-quality ASVs were obtained for subsequent analysis.

#### 2.6.2 Alpha and beta diversity analyses (*n* = 6 per group)

The feature table was rarefied to an even sampling depth (the minimum sequence count across all samples) to avoid biases due to varying sequencing depths. Alpha diversity indices (Chao1, Shannon, and Simpson) were then calculated using the q2-diversity plugin in QIIME2. Beta diversity was assessed based on Bray–Curtis distances and visualized using principal coordinates analysis (PCoA). The statistical significance of group separation in the PCoA was tested using permutational multivariate analysis of variance (PERMANOVA) with 999 permutations, implemented via the q2-diversity plugin.

#### 2.6.3 Differential abundance analysis (*n* = 6 per group)

Differential abundance analysis of microbial taxa at the genus level was performed using Analysis of Compositions of Microbiomes with Bias Correction (ANCOM-BC). This method accounts for the compositional nature of microbiome data and provides log-fold changes (effect sizes) and confidence intervals with false discovery rate (FDR) correction (*q*-value < 0.05 considered significant). For comparative purposes, linear discriminant analysis effect size (LEfSe) analysis was also conducted, with an LDA score threshold of 2.5.

### 2.7 Detection of fecal metabolites by LC–MS

#### 2.7.1 Metabolite extraction

Cecal contents were slowly thawed at 4 °C. Approximately 50 mg of each sample was precisely weighed and added to 400 μL of pre-cooled extraction solvent (methanol: water, 4:1 v/v) containing internal standards (L-2-chlorophenylalanine, 0.02 mg/mL). The mixture was homogenized using a frozen tissue grinder at −10 °C and 50 Hz for 6 min, followed by low-temperature ultrasonication (5 °C, 40 kHz) for 30 min. Samples were then incubated at −20 °C for 30 min and centrifuged at 13,000 × *g* for 15 min at 4 °C to pellet debris. The supernatant was transferred to a vial for LC-MS analysis. Additionally, equal volumes of supernatants from each sample were pooled to generate a quality control (QC) sample.

#### 2.7.2 LC-MS analysis (*n* = 6 per group)

Metabolite separation was performed using an Agilent 1290 Infinity UHPLC system equipped with a HILIC chromatographic column. The mobile phase consisted of (A) water containing 25 mM ammonium acetate and 25 mM formic acid (for positive ion mode) or acetic acid (for negative ion mode), and (B) acetonitrile (positive mode) or methanol (negative mode). The gradient elution program was as follows: 0–0.5 min, 95% B; 0.5–7 min, 95% to 65% B; 7–8 min, 65% to 40% B; 8–9 min, 40% B; 9–9.1 min, 40%–95% B; 9.1–12 min, 95% B. The flow rate was 0.5 mL/min, the column temperature was maintained at 25 °C, and the injection volume was 2 μL. Mass spectrometry detection was conducted using a Triple TOF 6600 mass spectrometer (AB SCIEX) with electrospray ionization (ESI) in both positive and negative ion modes. The key parameters were set as follows: ion source gas 1 and 2, 50 psi; curtain gas, 35 psi; source temperature, 500 °C; ion spray voltage, +5,500 V (positive mode) and −4,500 V (negative mode); declustering potential, 80 V; collision energy, 40 ± 20 eV; and scan range, m/z 50–1,200.

#### 2.7.3 Data preprocessing and metabolite identification

Raw data files were converted to mzXML format and processed using Progenesis QI software (v3.0, Waters Corporation) for peak picking, alignment, retention time correction, and peak area extraction, generating a data matrix comprising retention time, m/z values, and peak intensity. Metabolite identification was conducted by matching MS and MS/MS spectra against the HMDB^1^ and MetLin^2^ databases, with a mass tolerance set at 10 ppm for precursor ions. To ensure high-confidence identification, the initial set of identifications was filtered to include only those metabolites that were robustly detected across quality control (QC) samples. This process yielded a total of 590 metabolites for subsequent statistical analysis. According to the confidence levels defined by the Metabolomics Standards Initiative (MSI), the identified metabolites in this study were primarily at Level 2 (putative annotation).

#### 2.7.4 Multivariate statistical analysis and model validation (*n* = 6 per group)

The resulting data matrix was imported into MetaboAnalyst 5.0 for multivariate analysis. Orthogonal partial least squares discriminant analysis (OPLS-DA) was employed to maximize group separation. Model robustness was evaluated using 7-fold cross-validation and permutation tests (200 permutations). Models were considered valid if the *Q*^2^ value (predictive ability) of permuted models was significantly lower than that of the original model, confirming the absence of overfitting.

### 2.8 Statistical analysis

All data are presented as the mean ± standard deviation (SD). Normality of data distribution and homogeneity of variances were assessed using the Shapiro–Wilk and Levene’s tests, respectively. For comparisons among multiple groups, one-way analysis of variance (ANOVA) was performed, followed by Tukey’s honestly significant difference (HSD) test for *post-hoc* comparisons. Spearman’s correlation analysis was utilized to explore relationships among stress responses, gut microbiota, and metabolomic profiles. Intestinal microbiota data were subjected to alpha and beta diversity analyses using QIIME software, while R programming language was employed to generate visual representations of alpha diversity indices and principal coordinate analysis (PCoA) results. Species diversity analysis was performed using linear discriminant analysis effect size (LEfSe). In metabolomics analysis, univariate statistical methods (volcano plots) and multivariate statistical analyses, including principal component analysis (PCA) and OPLS-DA, were applied to investigate intestinal metabolites across rat groups. Key differential metabolites were further analyzed using MetaboAnalyst 5.0 for pathway analysis and compared with the Kyoto Encyclopedia of Genes and Genomes (KEGG) database to identify potentially affected metabolic pathways. All statistical analyses were conducted using SPSS software (version 26.0), and *p*-values < 0.05 were considered statistically significant. False discovery rate (FDR) correction was applied to all correlation analyses to account for multiple testing, with *q*-value < 0.05 considered statistically significant.

## 3 Results

### 3.1 Effects of policosanol on cell viability, ROS levels, and antioxidant activity in NCTC1469 cells

**Figure 2:**
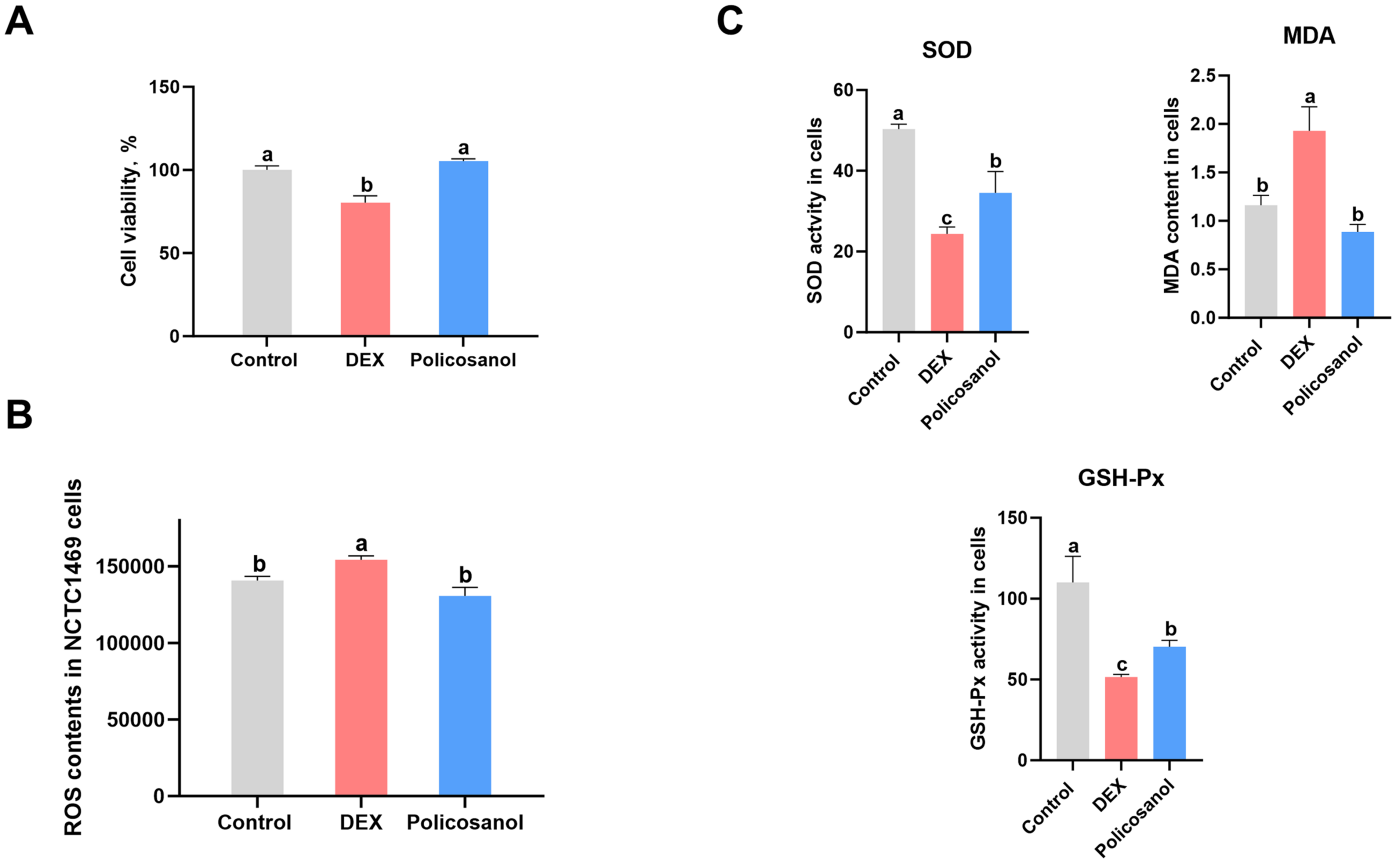
Evaluation of alterations in **(A)** cellular vitality, **(B)** reactive oxygen species (ROS) levels, and **(C)** key indicators of cellular oxidative stress, namely malondialdehyde (MDA), superoxide dismutase (SOD), and glutathione peroxidase (GSH-Px). Data are presented as means ± SEM (*n* = 6). Different letters (a, b) above bars indicate a statistically significant difference (*p* < 0.05). Groups that share a common letter are not significantly different.

Figure 2 illustrates the beneficial effects of policosanol on cell viability, reactive oxygen species (ROS) levels, and antioxidant function in NCTC1469 cells. Treatment with dexamethasone (DEX) significantly reduced cell viability (*p* < 0.05). However, supplementation with policosanol effectively reversed this inhibitory effect, significantly increasing cell viability compared to the DEX-treated group (*p* < 0.05). Similarly, policosanol significantly reduced the DEX-induced elevation in ROS levels (*p* < 0.05).

Regarding oxidative processes and antioxidant activities, DEX treatment significantly decreased the activities of SOD and GSH-Px, resulting in increased MDA levels, indicative of enhanced oxidation. However, policosanol administration mitigated these detrimental effects of DEX by restoring SOD and GSH-Px activities and reducing intracellular MDA levels. These results suggest that policosanol alleviates DEX-induced oxidative stress in NCTC1469 cells.

### 3.2 Effects of policosanol on rat growth performance, abdominal fat deposition, and hindlimb muscle weight

**Figure 3:**
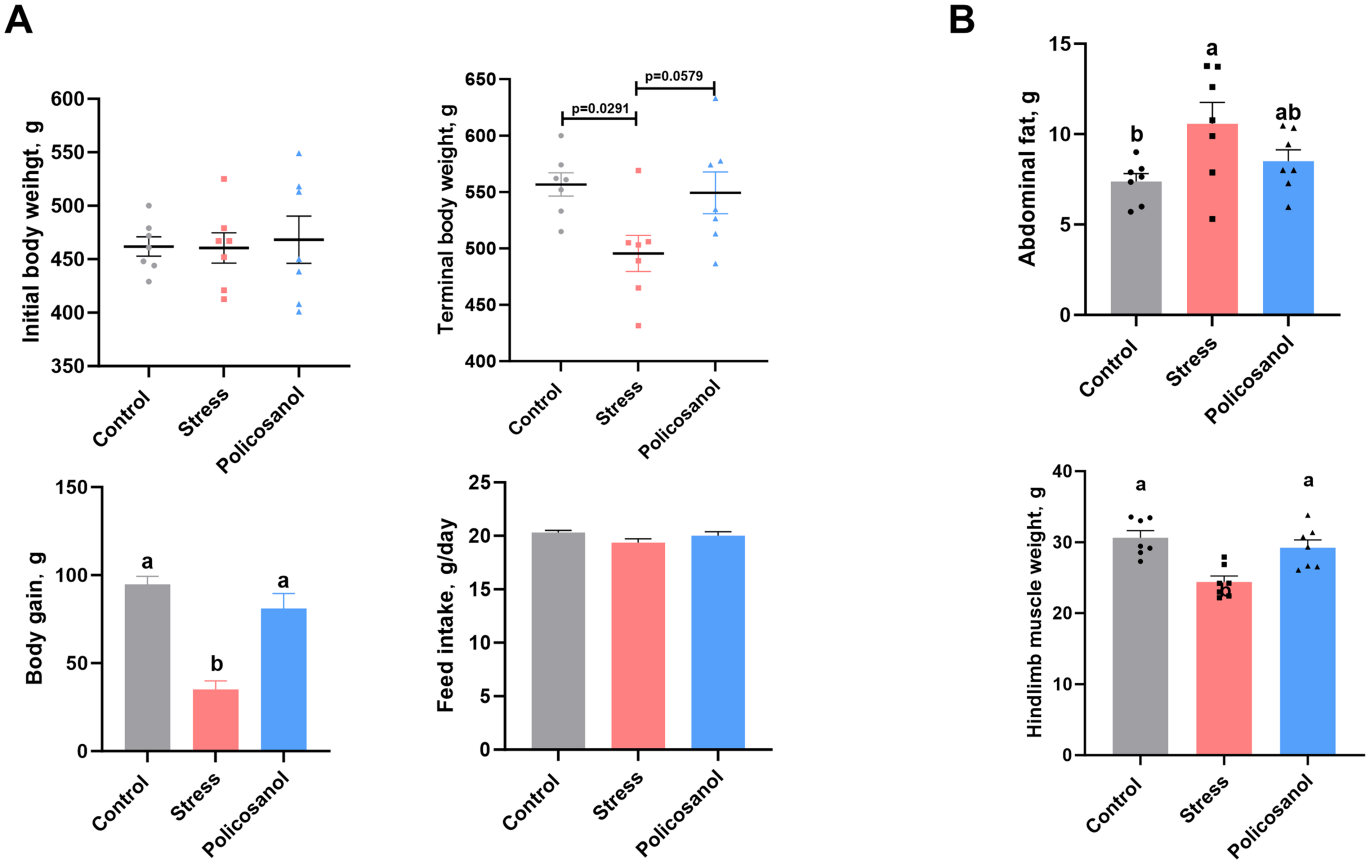
Effects of policosanol on rat production parameters. **(A)** Body weight, body weight gain, and feed intake. **(B)** Abdominal fat and hindlimb muscle weight. Data are reported as means ± SEM (*n* = 7). Different letters (a, b) above bars indicate a statistically significant difference (*p* < 0.05). Groups that share a common letter are not significantly different.

Figure 3 shows the effects of policosanol on rat growth performance, abdominal fat deposition, and hindlimb muscle weight. Compared with the control group, the stress group exhibited significant reductions in both final body weight and body weight gain (*p* < 0.05). However, supplementation with policosanol notably improved final body weight (*p* = 0.0579) and significantly increased body weight gain (*p* < 0.05). There were no significant differences in feed intake among the control, stress, and policosanol groups (*p* > 0.05).

As shown in Figure 3B, stress significantly increased abdominal fat deposition (*p* < 0.05) and reduced hindlimb muscle weight (*p* < 0.05) in rats. In contrast, policosanol supplementation significantly attenuated stress-induced abdominal fat accumulation and hindlimb muscle weight loss (*p* < 0.05). These findings suggest that policosanol improves growth performance and alleviates the adverse effects of stress on body composition in rats.

### 3.3 Effects of policosanol on serum cortisol, catecholamine, and antioxidative activity in serum and liver

**Figure 4:**
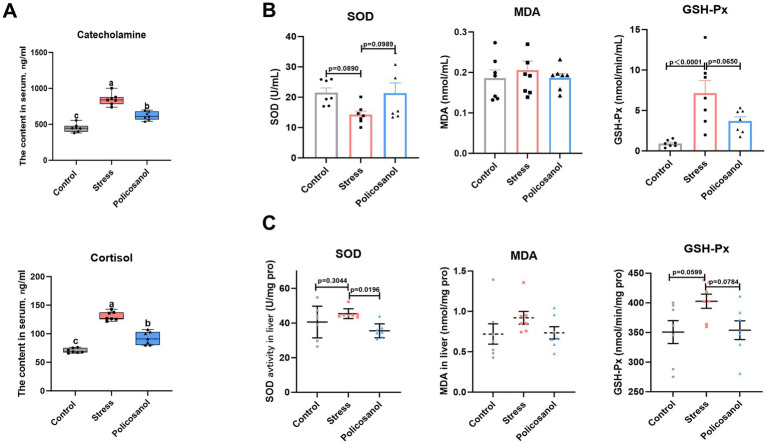
Impacts of policosanol on serum and liver indicators. **(A)** Serum catecholamine (CA) and cortisol levels. **(B)** Serum oxidative stress indicators (MDA, SOD, GSH-Px). **(C)** Hepatic oxidative stress indicators (MDA, SOD, GSH-Px). Data are reported as means ± SEM (*n* = 7). Different letters (a, b) above bars indicate a statistically significant difference (*p* < 0.05). Groups that share a common letter are not significantly different.

Figure 4 illustrates the modulation of serum cortisol, catecholamine, and antioxidant capacity following stress exposure and policosanol treatment. Stress significantly elevated serum cortisol and catecholamine concentrations (*p* < 0.05). Policosanol supplementation significantly reduced serum cortisol and catecholamine levels (*p* < 0.05), although these remained slightly elevated compared to the control group.

Regarding serum antioxidant enzyme activities, stress caused a decrease in SOD activity (*p* = 0.0890) and a significant increase in GSH-Px activity (*p* < 0.05). Policosanol treatment effectively attenuated these stress-induced changes in SOD and GSH-Px. In the liver, stress tended to increase GSH-Px levels (*p* = 0.0599) but did not significantly alter SOD and GSH-Px activities (*p* > 0.05). However, policosanol supplementation resulted in significantly lower SOD activity (*p* < 0.05) and a marginal reduction in GSH-Px activity (*p* = 0.0784) compared with the stress group. Notably, no significant differences in MDA levels were observed in serum or liver tissues among the control, stress, and policosanol groups (*p* > 0.05). These findings suggest that policosanol can regulate stress hormone levels and antioxidant enzyme activities, potentially contributing to its anti-stress effects.

### 3.4 Effects of policosanol on gut microbiota

**Figure 5:**
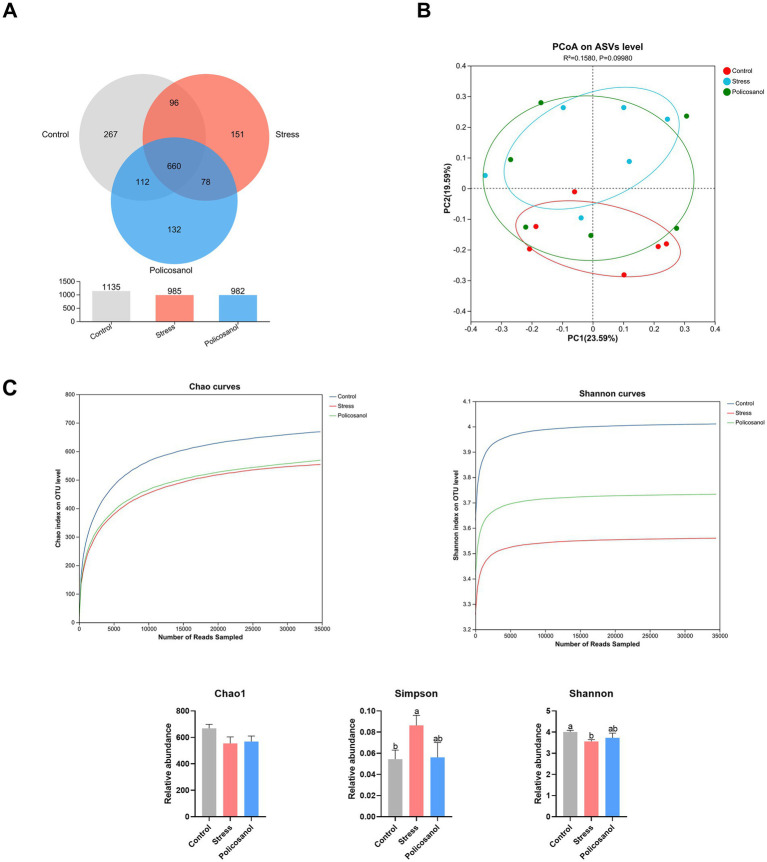
Influence of policosanol on gut microbiota abundance and diversity. **(A)** Venn diagram of amplicon sequence variants (ASVs) shared among groups. **(B)** Principal coordinates analysis (PCoA) based on Bray–Curtis distances showing differentiation trends among groups (PERMANOVA, *p* = 0.0998). **(C)** Rarefaction curves and alpha diversity indices (Shannon and Simpson). Each group consisted of six replicates (*n* = 6). The average sequencing depth was 65,974 reads per sample (total reads: 1,187,528). Statistical analysis of differential abundance was performed using ANCOM-BC (FDR *q* < 0.05).

Figure 5 illustrates the effects of policosanol on gut microbiota abundance and diversity. A total of 3,102 operational taxonomic units (ASVs) were identified across the three groups. The control, stress, and policosanol groups contained 1,135, 985, and 982 ASVs, respectively. Among these ASVs, 660 were shared across all groups, while the control, stress, and policosanol groups had 267, 151, and 132 unique ASVs, respectively (Figure 5A). Alpha diversity analysis (Figure 5C) showed that the rarefaction curves for Chao1 and Shannon indices plateaued with increasing sequencing depth, indicating sufficient coverage to represent microbial diversity. Stress significantly reduced the Shannon index (*p* < 0.05) and increased the Simpson index (*p* < 0.05). However, policosanol treatment effectively reversed these stress-induced changes by increasing the Shannon index and decreasing the Simpson index. Principal coordinates analysis (PCoA) for beta diversity (Figure 5B) revealed differentiation trends among groups (Adonis, *p* = 0.0998). Samples from the control group were positioned primarily in the lower quadrants, while the stress group occupied the upper quadrants, with the policosanol group distributed across all quadrants. The control and stress groups were distinctly separated, with minimal overlap.

**Figure 6:**
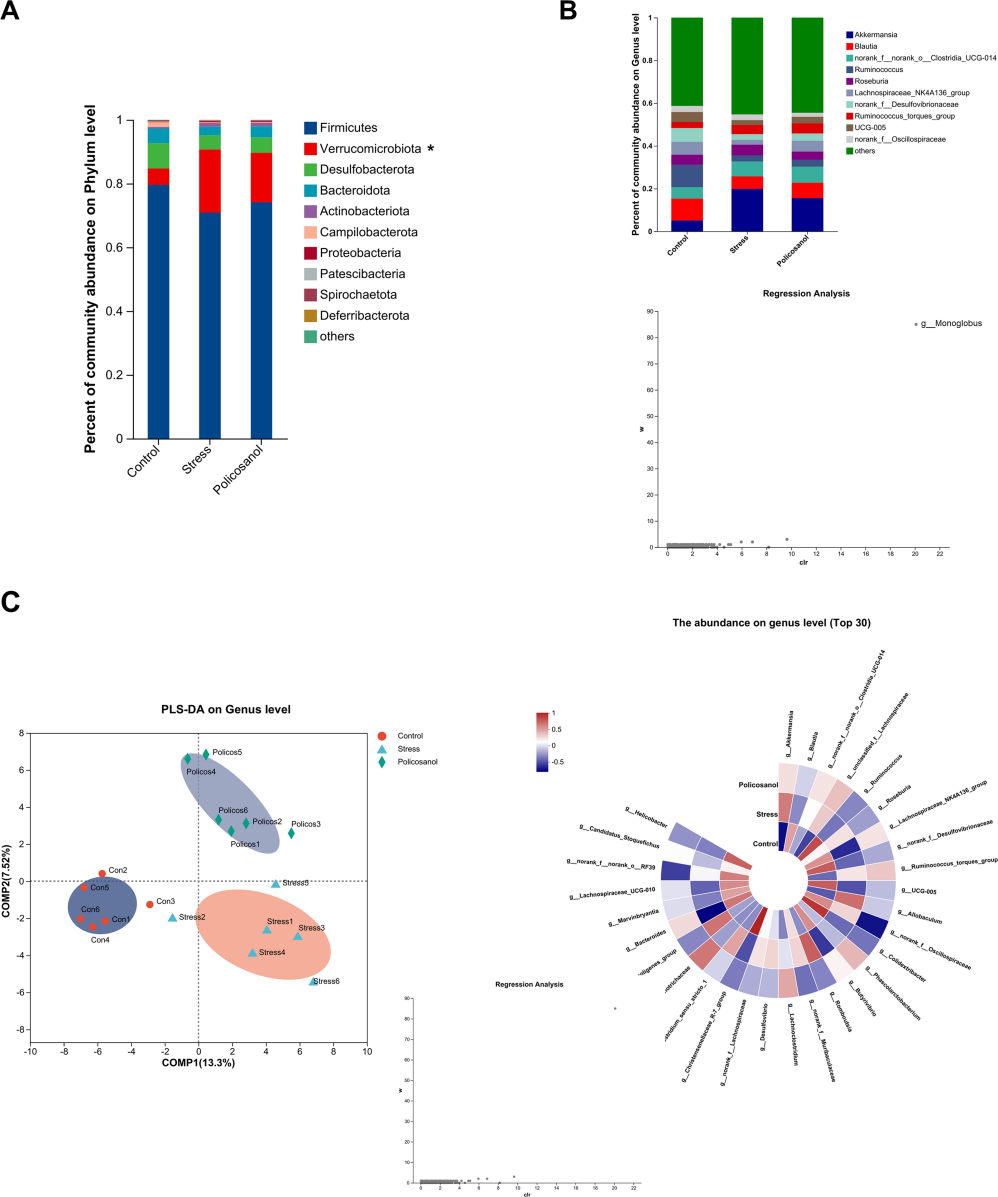
Gut microbiota composition and differential abundance analysis. **(A)** Relative abundance of microbial communities at the phylum level. **(B)** Relative abundance at the genus level (top 10 genera shown). ANCOM-BC analysis identified g__Monoglobus as the only significantly differentially abundant genus (*W* = 85, FDR *q* < 0.05), though it is not among the top 10 most abundant genera shown here. **(C)** Partial least squares discriminant analysis (PLS-DA) at the genus level. Each group consisted of six replicates (*n* = 6). Total sequencing reads: 1,187,528 across 18 samples. Differential abundance analysis using ANCOM-BC (FDR *q* < 0.05) identified g__Monoglobus as the only significantly differentially abundant genus (*W* = 85).

Figure 6 presents microbial composition and interspecies analyses. At the phylum level, Firmicutes, Verrucomicrobiota, and Desulfobacterota were dominant, comprising over 90% of the gut microbiota. Compared with controls, stress significantly elevated the relative abundance of Verrucomicrobiota (*p* < 0.05), whereas policosanol treatment effectively reduced this stress-induced increase. At the genus level, the four most abundant genera across all groups were Akkermansia, Blautia, norank_f_norank_o_Clostridia_UCG-014, and Ruminococcus. Stress significantly increased the proportion of Akkermansia within Verrucomicrobiota (*p* < 0.05), which was effectively mitigated by policosanol intervention. Using linear discriminant analysis effect size (LEfSe) with an LDA threshold of 2.5, a total of 47 potential biomarkers were identified in an exploratory analysis (Supplementary Figure S3). However, it should be noted that LEfSe does not account for the compositional nature of microbiome data. The statistically rigorous ANCOM-BC analysis identified only g__Monoglobus as significantly differentially abundant after FDR correction. Additionally, partial least squares discriminant analysis (PLS-DA) at the genus level (Figure 6C) showed clear separation without overlap among the control, stress, and policosanol groups. These findings indicate that policosanol can effectively modulate the composition and diversity of gut microbiota in rats under stress.

### 3.5 Effects of policosanol on gut metabolites

**Figure 7:**
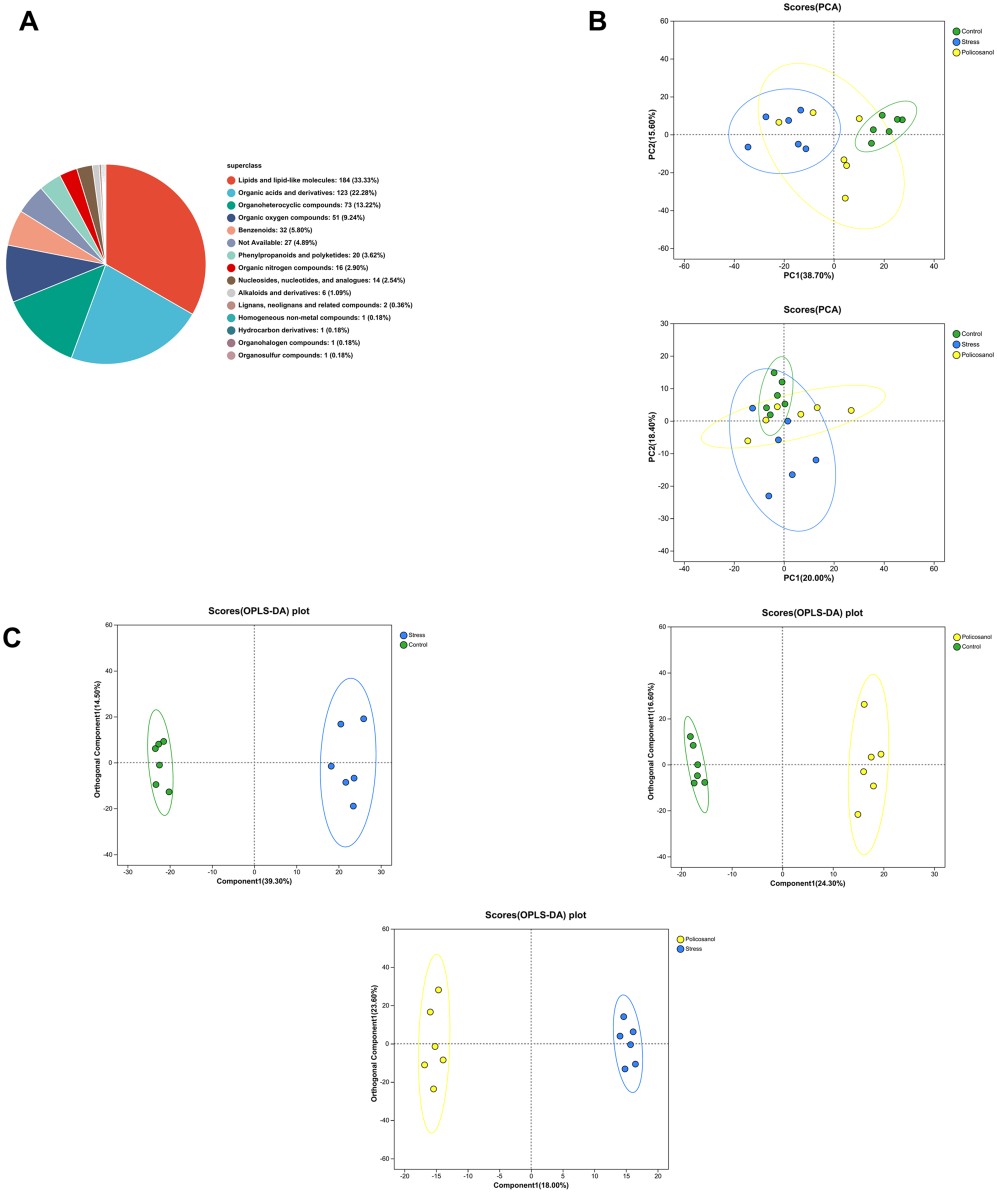
Classification and variation of metabolites. **(A)** Proportion of metabolites in each chemical class. **(B)** Principal component analysis (PCA) scores in positive and negative ion modes. **(C)** Orthogonal partial least squares discriminant analysis (OPLS-DA) scores for each comparative group. Each group consisted of six replicates (*n* = 6).

Figure 7 illustrates the classification and variation of metabolites among groups. All identified metabolites were classified according to chemical categories for statistical analysis (Figure 7A). Lipids and lipophilic molecules were the most abundant (33.33%), followed by organic acids and derivatives (22.28%), organoheterocyclic compounds (13.22%), and organic oxygen compounds (9.24%). Other notable metabolite classes included benzenoids, phenylpropanoids, and polyketides. A principal component analysis (PCA) was performed to visualize overall trends (Figure 7B). The PCA model (mixed ion mode) showed modeling explanation rates (*R*^2^X) of 0.656 (Stress vs. Control), 0.637 (Policosanol vs. Stress), and 0.641 (Policosanol vs. Control). In positive ion mode, samples from each group clustered distinctly within the 95% confidence interval, clearly differentiating among groups, while separation was less pronounced in negative ion mode. To further examine metabolic differences, orthogonal partial least squares-discriminant analysis (OPLS-DA) was conducted. The OPLS-DA scores (Figure 7C) in mixed ion mode demonstrated tight clustering and clear separation among groups. Validation parameters (*R*^2^X, *R*^2^Y, and *Q*^2^) were 0.538, 0.993, and 0.883 (Stress vs. Control); 0.652, 0.997, and 0.701 (Policosanol vs. Stress); and 0.621, 0.995, and 0.865 (Policosanol vs. Control), respectively, indicating robust reliability of the models.

**Figure 8:**
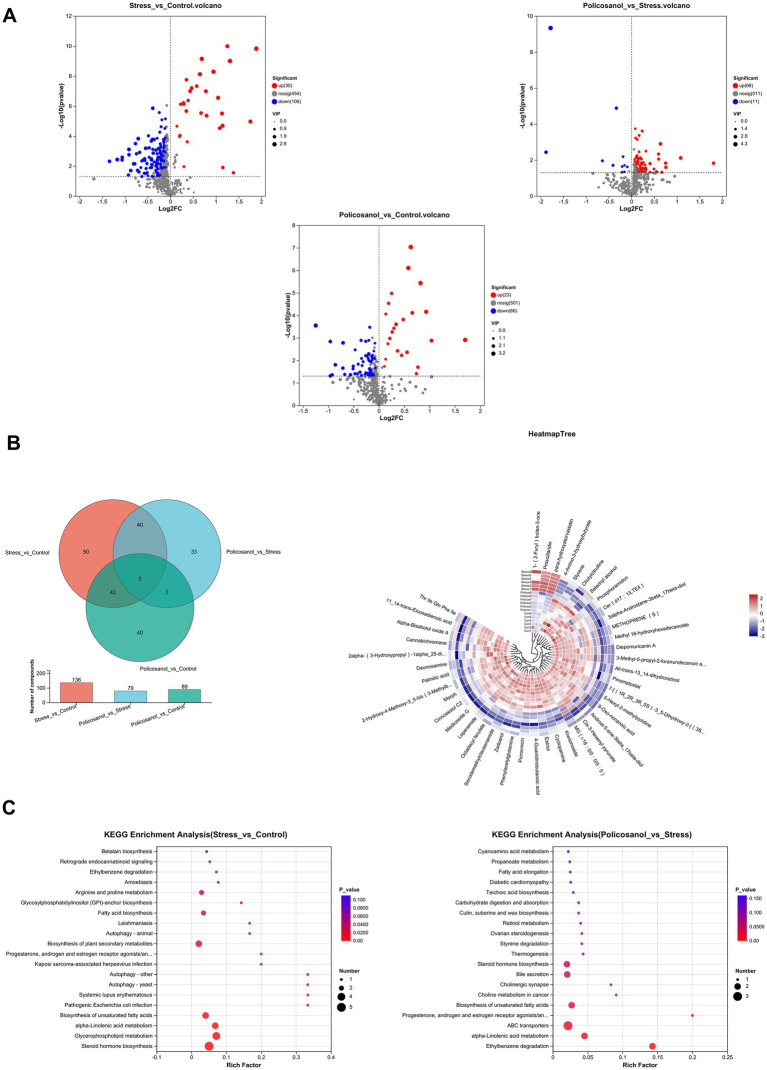
Inter-group differences in metabolites. **(A)** Volcano plots of differential metabolites. **(B)** Differential metabolites among three groups. **(C)** Scatter plot of KEGG pathway enrichment analysis. Each group consisted of six replicates (*n* = 6).

The differences in metabolites among groups were further investigated (Figure 8). The volcano plot (Figure 8A) indicated that, compared with the control group, the stress group had 30 significantly upregulated and 106 significantly downregulated metabolites. Comparing the policosanol group with the stress group identified 68 upregulated and 11 downregulated metabolites, while comparing the policosanol group with the control group revealed 23 upregulated and 66 downregulated metabolites. Selected differential metabolites are highlighted in Figure 8B. Between the stress and control groups, metabolites exhibiting significant differences (*p* < 0.05) included Proscillaridin, 11,14-trans-Eicosadienoic acid, and Phenylacetylglutamine (PAGln). Proscillaridin levels were significantly elevated in the stress group, whereas levels of 11,14-trans-Eicosadienoic acid and PAGln were significantly decreased. Policosanol treatment reversed these trends, restoring metabolite levels toward control values. The scatter plot (Figure 8C) of KEGG pathway enrichment analysis revealed that steroid hormone biosynthesis, alpha-linolenic acid metabolism, and biosynthesis of unsaturated fatty acids were significantly enriched pathways (*p* < 0.05), closely associated with stress-response modulation, oxidative balance, and growth performance. Collectively, these findings suggest that policosanol can partially mitigate stress-induced disruptions of gut metabolites, thereby enhancing metabolic equilibrium and stress resilience in rats. Each analysis was conducted with six biological replicates (*n* = 6).

**Figure 9:**
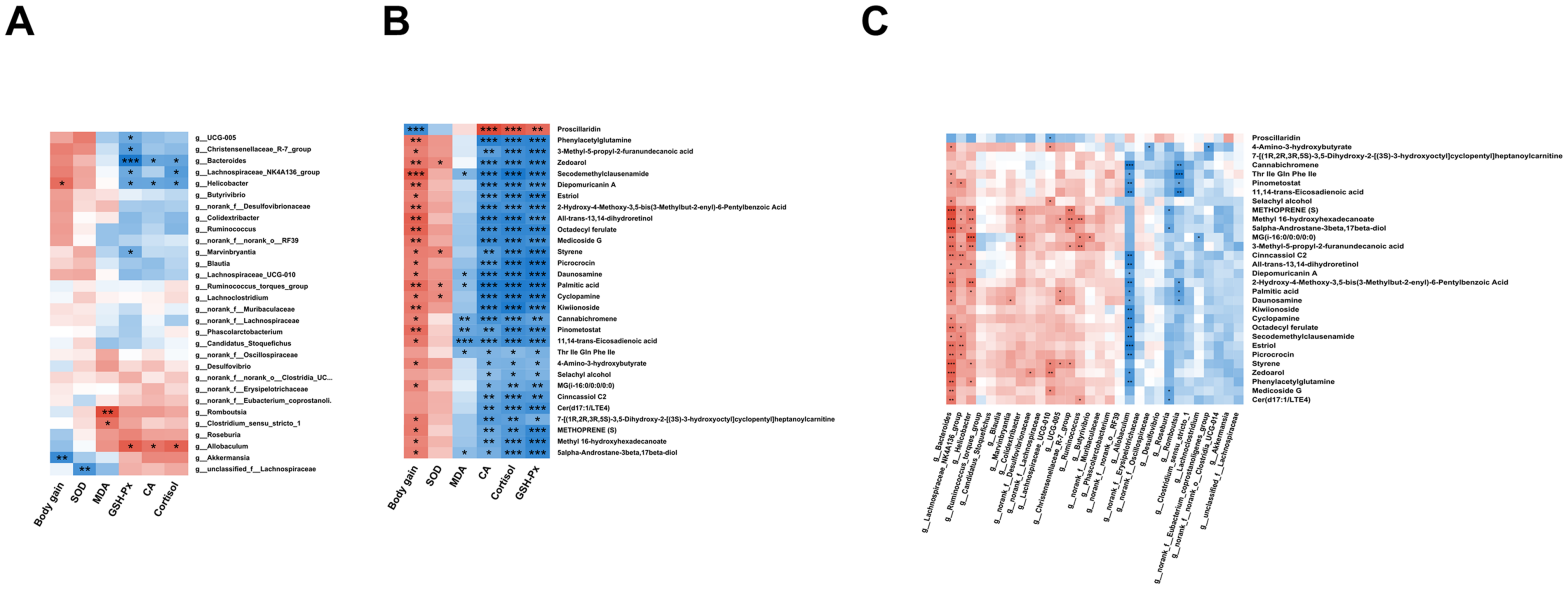
Correlation networks among growth performance, gut microbiota, and metabolites. **(A)** Correlation between phenotypic markers (Body Weight, Serum CA, Cortisol, MDA, SOD, GSH-Px) and gut microbiota. Total correlations tested: 180; FDR-significant correlations shown: 19. **(B)** Correlation between phenotypic markers and differential metabolites. Total correlations tested: 180; FDR-significant correlations shown: 128. **(C)** Correlation between gut microbiota and metabolites. Total correlations tested: 900; FDR-significant correlations shown: 104. Each group consisted of six replicates (*n* = 6). Significant correlations in the heatmaps are denoted by asterisks (*, FDR < 0.05; **, FDR < 0.01; ***, FDR < 0.001). Only correlations with FDR-corrected *q*-value < 0.05 are displayed. Red and blue squares represent positive and negative correlations, respectively, with color intensity corresponding to the absolute value of the correlation coefficient.

Correlations among growth performance, gut microbiota, and gut metabolites were also analyzed (Figure 9). In Figure 9A, analysis of the relationship between growth performance and gut microbiota indicated that the genus positively correlated with body weight gain was *g_Helicobacter* (*p* < 0.05), while the negatively correlated genus was *g_Akkermansia* (*p* < 0.05). Genera positively associated with antioxidant capacity included *g_Allobaculum* (*p* < 0.05), whereas those negatively correlated comprised *g_unclassified_f_Lachnospiraceae*, *g_Romboutsia*, and *g_Bacteroides* (*p* < 0.05). Regarding stress-related hormones, the genus positively correlated was *g_Allobaculum* (*p* < 0.05), while negatively correlated genera included *g_Bacteroides* and *g_Helicobacter* (*p* < 0.05). Figure 9B shows the correlations between growth performance and metabolites. Metabolites negatively associated with body weight gain included Proscillaridin (*p* < 0.05), whereas significantly positively associated metabolites consisted of 25 species, including Secodemethylclausenamide and PAGln (*p* < 0.05). Regarding antioxidant capacity-related metabolites, GSH-Px positively correlated with Proscillaridin (*p* < 0.05), while negatively correlating with Estriol, Methyl 16-hydroxyhexadecanoate, and 5alpha-androstane-3beta, 17beta-diol (*p* < 0.05). Additionally, MDA displayed significant negative correlations with 11,14-trans-Eicosadienoic acid and Cannabichromene (*p* < 0.05), whereas SOD exhibited significant positive correlations with Zedoarol and Palmitic acid (*p* < 0.05). Regarding stress hormone secretion, Proscillaridin showed a positive correlation (*p* < 0.05), whereas Cannabichromene, 11,14-trans-Eicosadienoic acid, PAGln, and Estriol were negatively correlated (*p* < 0.05). Finally, Figure 9C presents the correlations between gut microbiota and metabolites. *g_Bacteroides* significantly positively correlated with Methyl 16-hydroxyhexadecanoate, 5alpha-androstane-3beta,17beta-diol, PAGln, Zedoarol, and other metabolites (*p* < 0.05). Moreover, *g_Helicobacter* displayed significant positive correlations with MG (i-16:0/0:0/0:0), Cis-3-Hexenyl pyruvate, and Methyl 16-hydroxyhexadecanoate (*p* < 0.05), whereas *g_Allobaculum* exhibited negative correlations with Cannabichromene, 11,14-trans-Eicosadienoic acid, PAGln, and Estriol (*p* < 0.05). Lastly, *g_Romboutsia* negatively correlated with Cannabichromene and 11,14-trans-Eicosadienoic acid (*p* < 0.05).

## 4 Discussion

### 4.1 Stress-induced impairment of growth performance, gut microbiota, and metabolomics in rats

Stress is a complex physiological and psychological response elicited by diverse internal and external stimuli (13, 14). Excessive stress can profoundly affect both mental and physical health (15). Psychologically, it may lead to anxiety, depression, and other mental health disorders (16). Physiologically, stress can disrupt immune function, metabolism, and cardiovascular health, increasing susceptibility to various diseases (17).

The body’s stress response is primarily regulated by the neuroendocrine axis [hypothalamic-pituitary-adrenal (HPA) axis] (18). Activation of this axis results in the release of stress hormones, such as adrenal corticosteroids and CA, which modulate multiple physiological processes, including metabolism, cardiovascular function, and immunity, thereby facilitating adaptation to stress (19). Oxidative stress is also an essential component of the stress response (20). Under stress conditions, cells produce reactive oxygen species (ROS) and other oxidizing substances (e.g., MDA), disrupting antioxidant enzyme activities (SOD and GSH-Px) and exacerbating oxidative damage (21). In this study, DEX treatment of NCTC1469 cells significantly increased intracellular ROS and MDA levels while decreasing SOD and GSH-Px activities, indicating that DEX successfully induced an imbalance in the oxidative-antioxidant system, establishing an effective cellular stress model.

Previous studies have demonstrated that prolonged stress results in excessive energy consumption and redistribution, leading to body wasting, muscle loss, and abdominal fat deposition (22–24). Consistent with these findings, our study showed that stress treatment activated the HPA axis, increased catecholamine and cortisol release, altered antioxidant capacity by modulating SOD and GSH-Px activities in serum and liver, reduced body weight gain and hindlimb muscle mass, and increased abdominal fat deposition. However, unlike in the cell experiments, stress enhanced GSH-Px activity in serum and liver tissues. This discrepancy might be due to increased biosynthesis of GSH-Px *in vivo* to maintain oxidative balance under external stress conditions (25, 26), suggesting that our rat model effectively simulated stress responses.

High-throughput 16S rRNA sequencing revealed that stress decreased the abundance and diversity of cecal microbiota. Notably, at the phylum level, stress significantly increased the relative abundance of p_Verrucomicrobiota, indicating disrupted structural integrity of gut microbiota. At the genus level, the stress group exhibited higher abundances of *g_Akkermansia*, *g_Allobaculum*, and *g_Romboutsia*, whereas the control group had higher proportions of *g_Bacteroides*, *g_Ruminococcus*, and *g_Helicobacter*. *g_Bacteroides*, a commensal microorganism, is a crucial genus within gut microbiota, supporting host health through symbiotic interactions (27, 28). Prior research has indicated that stress-induced shifts in the abundance of *g_Akkermansia* and *g_Allobaculum* could elevate oxidative stress (29, 30). Thus, stress appears to disrupt microbiota balance by increasing harmful bacterial populations and reducing beneficial taxa. However, the stress-induced increase in *g_Akkermansia* observed in our study contrasts with previous reports of decreased abundance under chronic stress conditions (29, 30). Such discrepancies are common in microbiome research and can be attributed to several factors. Firstly, the specific nature of the stressor is critical; our chronic restraint stress model may differentially influence gut microbial composition compared to paradigms like social defeat or unpredictable mild stress employed in other studies. Secondly, host-specific factors, including genetic background, baseline microbiota composition, and diet, profoundly affect microbial responses to the same stressor. The highly context-dependent behavior of *Akkermansia muciniphila* has been documented, with its effects ranging from beneficial to potentially harmful depending on the host’s physiological or pathological state (31). Therefore, our findings emphasize that microbial responses to stress are not uniform but rather shaped by complex interactions among experimental and host variables. This underscores the importance of clearly reporting experimental conditions and exercising caution against generalizing microbial responses across diverse experimental models.

Metabolomics involves the comprehensive detection and quantitative analysis of metabolites in biological samples, examining dynamic metabolic changes in response to specific stimuli. Using LC–MS-based untargeted metabolomics, we identified significantly lower levels of 4-Guanidinobutanoic acid and 11,14-trans-Eicosadienoic acid, both associated with organismal health, in the stress group compared with controls, whereas levels of Proscillaridin were significantly higher. Previous studies have confirmed that stress-induced changes in gut microbial composition can reduce cecal concentrations of beneficial organic acids, such as 4-Guanidinobutanoic acid and 11,14-trans-Eicosadienoic acid (32, 33). Supplementation with 4-Guanidinobutanoic acid (GAA) reportedly promotes animal growth (34), whereas Proscillaridin has demonstrated effects such as inhibiting cell proliferation, migration, and invasion, and inducing apoptosis and autophagy, thereby potentially impairing growth and development (35). Collectively, these findings suggest that stress influences animal growth and health by altering gut microbiota composition and metabolite profiles.

### 4.2 Enhancement of growth performance, gut microbiota, and metabolomics by policosanol supplementation

Policosanol has been reported to promote animal health and growth through various mechanisms, including lipid-lowering effects, elevation of high-density lipoprotein cholesterol (HDL-C), antioxidant and anti-inflammatory activities, and facilitation of tissue regeneration (5–10). Consistent with these studies, our results demonstrated that policosanol significantly decreased cortisol levels in stressed rats and enhanced cellular antioxidant capacity by reducing oxidative stress markers such as ROS and MDA, as well as modulating SOD and GSH-Px activities, thereby alleviating stress responses.

The body’s oxidative and antioxidant systems maintain a dynamic equilibrium. Under stress, antioxidant enzymes like SOD and GSH-Px are upregulated to scavenge free radicals and counteract oxidative damage (25, 26). Our findings indicated that policosanol supplementation reduced the stress-induced increase in GSH-Px activity in serum and liver, thus preserving the oxidative-antioxidant balance. These results highlight the beneficial role of policosanol in mitigating stress-induced hormonal and oxidative disruptions.

Previous studies have shown that policosanol reduces fat accumulation and inhibits weight gain in animals fed high-fat diets (4, 5, 36). In BV-2 microglial cell lines treated with recombinant high-density lipoprotein (rHDL) combined with policosanol, policosanol promoted cell growth and replication while suppressing apoptosis (10). Similarly, our study found that policosanol supplementation significantly reduced abdominal fat deposition, increased muscle mass, and improved body weight compared with the stress group, suggesting its potential to reverse stress-induced declines in productivity.

Gut microbiota composition is dynamically influenced by genetics, environment, health status, and diet. Policosanol supplementation has been reported to mitigate high-fat diet-induced microbiota restructuring, suppress harmful bacteria proliferation, and promote beneficial bacteria growth (37, 38). Our findings showed that policosanol supplementation alleviated stress-induced reductions in alpha diversity (increased Shannon index and decreased Simpson index). At the phylum level, policosanol significantly decreased the abundance of Verrucomicrobiota, while at the genus level, it reduced *g_Akkermansia*, *g_Allobaculum*, and *g_Romboutsia* abundances and increased those of beneficial bacteria, including *g_Bacteroides*, *g_Ruminococcus*, and *g_Helicobacter*. These results suggest that policosanol can rectify stress-induced gut microbiota disruptions, enhance beneficial bacterial populations, and consequently support animal growth and health.

Using LC-MS-based untargeted metabolomics, we identified 11,14-trans-Eicosadienoic acid, Estriol, and Secodemethylclausenamide as key metabolites distinguishing the policosanol group from the stress group. 11,14-trans-Eicosadienoic acid, a polyunsaturated fatty acid, can reduce intestinal inflammation, improve nutrient absorption and utilization, and enhance growth rate and feed efficiency in rats (39). Additionally, low-dose Estriol treatment has been shown to mitigate oxidative stress, enhance immune function, and support growth in mice (40). Our study demonstrated that policosanol supplementation significantly increased intestinal levels of 11,14-trans-Eicosadienoic acid and Estriol compared with the stress group. These differential metabolites may represent critical factors underlying observed improvements in growth and health status.

Pathway analysis of selected differential metabolites identified three significantly enriched metabolic pathways: steroid hormone biosynthesis, alpha-linolenic acid metabolism, and biosynthesis of unsaturated fatty acids. The steroid hormone biosynthesis pathway is crucial for physiological functions in animals, regulating reproductive processes, growth, and development, and is associated with certain diseases. Influenced by the gut microbiota, biosynthesis of unsaturated fatty acids (UFAs) plays an essential role in maintaining intestinal homeostasis. UFAs serve as components of cell membranes and participate in signal transduction and inflammatory response regulation. Alpha-linolenic acid (ALA), an essential ω-3 polyunsaturated fatty acid, can be enzymatically converted into longer-chain ω-3 polyunsaturated fatty acids. Activation of ALA metabolism can reduce inflammatory factor production through signaling pathways such as PPARα (41, 42), thereby alleviating inflammation, decreasing oxidative stress, and protecting cells from free radical damage. Thus, alpha-linolenic acid metabolism is significant for maintaining cellular function and disease prevention.

### 4.3 Associations of gut microbiota and its metabolites with rat growth and health

The gut microbiota comprises approximately 500–1,000 species and roughly 100 trillion bacteria, about 10 times the number of host cells. It plays essential roles in host physiology, metabolism, and immune regulation, significantly influencing host health. Factors such as age, diet, antibiotic use, and psychological stress can affect its composition and diversity, and different bacterial groups have varying effects on growth and health.

The abundance of *g_Akkermansia* is closely associated with host health, and higher levels are linked to compromised intestinal barrier integrity and exacerbated inflammatory responses (43). Animal studies have indicated that certain *g_Helicobacter* species may possess anti-inflammatory properties, alleviating inflammatory disease symptoms and reducing the incidence and progression of gastritis (44). *g_Bacteroides* can metabolize complex carbohydrates, modulate immune responses, and inhibit pathogen colonization, thus playing a significant role in maintaining intestinal health and promoting growth (45, 46). Our study revealed a significant negative correlation between *g_Akkermansia* and body weight gain. Additionally, *g_Helicobacter* and *g_Bacteroides* negatively correlated with serum corticosterone and CA, respectively. *g_Helicobacter* was positively correlated with body weight gain and negatively correlated with serum GSH-Px. These findings indicate positive associations of *g_Bacteroides* and *g_Helicobacter* with growth and development in rats.

Gut microbiota-derived metabolites also correlate with animal growth and health. Previous research demonstrated that the gut metabolite Proscillaridin can induce intestinal inflammation, increase intestinal permeability, impair barrier function, and thus negatively impact growth and feed conversion efficiency in mice (47). In contrast, PAGln, a microbial metabolite, promotes immune cell activation, enhances immune responses, and improves animal growth status and stress resistance under adverse conditions (48). Our results showed close relationships between various metabolites and body weight gain, GSH-Px, CA, and cortisol. Specifically, Proscillaridin negatively correlated with body weight gain but positively correlated with stress hormone secretion and GSH-Px activity. Conversely, 11,14-trans-Eicosadienoic acid and PAGln positively correlated with body weight gain and negatively correlated with stress hormone secretion and GSH-Px activity. These results suggest negative correlations of growth and health with Proscillaridin and positive correlations with 11,14-trans-Eicosadienoic acid and PAGln.

Despite the lack of significant overall changes in the abundance of g_*Bacteroides* across groups as determined by ANCOM-BC, our multi-omics correlation analysis revealed a strong positive association between its relative abundance and the levels of phenylacetylglutamine (PAGln) at the individual level (Figure 9C). This suggests that the anti-stress effects of policosanol may be mediated not by altering the population size of g_*Bacteroides*, but rather by modulating its functional output, specifically the production of PAGln. PAGln, a microbial metabolite with recognized immunomodulatory and potential anti-stress properties (48), was itself correlated with improved growth performance and reduced stress hormones (Figure 9B). Therefore, the identified g_*Bacteroides*–PAGln functional axis provides a novel perspective on policosanol’s mechanism of action: it may exert its benefits by optimizing the metabolic output of specific beneficial bacteria, rather than through non-specific changes in their abundance. This finding underscores that in assessing microbial interventions, focusing on microbial metabolic activity can be as crucial as tracking compositional shifts.

### 4.4 Limitations and future perspectives

Although our study identified a significant correlation between *g_Bacteroides* abundance, elevated PAGln levels, and improvement in stress-related phenotypes, the current experimental design does not permit conclusions regarding causality. The hypothesized *g_Bacteroides*–PAGln axis, while strongly suggested by our findings, requires validation through targeted mechanistic studies. Future research employing fecal microbiota transplantation (FMT) from policosanol-treated donors into germ-free or antibiotic-treated stressed recipients could conclusively determine whether microbiota changes alone can reproduce observed metabolic and physiological benefits. Additionally, direct supplementation of stressed animals with purified PAGln could help clarify whether this metabolite independently mediates anti-stress effects, separate from microbial alterations. Such interventional studies will be essential to move beyond correlation and definitively establish causality within this promising microbiota–metabolite–host axis.

Additionally, it should be noted that the present study did not include a policosanol-only control group, which limits our ability to discern the direct effects of policosanol in the absence of stress. Future studies incorporating such a group would help clarify whether policosanol exerts beneficial effects independently of stress conditions. And the exclusive use of male rats in our study represents a limitation, as sex-specific responses to stress and policosanol treatment remain to be investigated.

## 5 Conclusion

This study demonstrated that policosanol, a bioactive compound derived from rice bran wax, effectively alleviates chronic stress-induced growth deficits in rats by modulating gut microbiota composition and restoring metabolic homeostasis. Chronic stress significantly inhibited weight gain, elevated cortisol and catecholamine levels, and induced gut dysbiosis characterized by increased abundances of p_Verrucomicrobiota and *g_Akkermansia*, alongside disrupted metabolite profiles (e.g., elevated Proscillaridin, reduced PAGln). Policosanol supplementation reversed these effects, promoting weight gain, reducing cortisol and catecholamine secretion, and normalizing microbial composition (particularly p_Verrucomicrobiota) and metabolite concentrations (e.g., PAGln).

Notably, *g_Akkermansia* abundance correlated negatively with weight gain, whereas PAGln correlated positively with growth performance and negatively with GSH-Px, cortisol, and catecholamine levels. The coregulation of the gut microbiota and metabolome was highlighted by a strong correlation between *g_Bacteroides* and Phenylacetylglutamine (PAGln), suggesting a potential functional interaction that may contribute to the anti-stress effects of policosanol. Notably, this interaction was evident without a significant shift in the overall abundance of *g_Bacteroides*, implying that policosanol may modulate the metabolic activity of this bacterium rather than its proliferation.

These findings provide mechanistic insights into how policosanol mitigates stress via gut microbiota–metabolite interactions, laying a foundation for microbiota-targeted therapeutic strategies for stress-related disorders. Future research should focus on optimizing policosanol dosage and evaluating its long-term efficacy across various physiological contexts.

## Data Availability

The datasets presented in this study can be found in online repositories. The names of the repository/repositories and accession number(s) can be found in the article/Supplementary material.

## Footnotes

1
https://www.hmdb.ca/

2
https://metlin.scripps.edu/
