# Supplementary material for: Policosanol alleviates chronic stress-induced growth impairment via gut microbiota-metabolite interactions: insights from 16S rRNA sequencing and LC-MS metabolomics
Source: Front Nutr. 2026 Jan 5;12:1685003. doi: 10.3389/fnut.2025.1685003 (PMC12812650; doi:10.3389/fnut.2025.1685003)

**Figure legend**

Figure 1. Chromatographic profile and compositional analysis of policosanol derived from rice bran wax. Qualitative analysis of the main components in policosanol samples was conducted using high-grade alcohols as reference standards. The mass percentage content of each peak was calculated utilizing the normalization method. The policosanol mixture used in this study had a total content of long-chain alcohols (C22-C30) of >84% (by mass), with octacosanol (C28) as the predominant constituent.

Figure 2. Evaluation of alterations in (A) cellular vitality, (B) reactive oxygen species (ROS) levels, and (C) key indicators of cellular oxidative stress, namely malondialdehyde (MDA), superoxide dismutase (SOD), and glutathione peroxidase (GSH-Px). Data are presented as means ± SEM (n = 6). Different letters (a, b) above bars indicate a statistically significant difference (p < 0.05). Groups that share a common letter are not significantly different.

Figure 3. Effects of policosanol on rat production parameters. (A) Body weight, body weight gain, and feed intake. (B) Abdominal fat and hindlimb muscle weight. Data are reported as means ± SEM (n = 7). Different letters (a, b) above bars indicate a statistically significant difference (p < 0.05). Groups that share a common letter are not significantly different.

Figure 4. Impacts of policosanol on serum and liver indicators. (A) Serum catecholamine (CA) and cortisol levels. (B) Serum oxidative stress indicators (MDA, SOD, GSH-Px). (C) Hepatic oxidative stress indicators (MDA, SOD, GSH-Px). Data are reported as means ± SEM (n = 7). Different letters (a, b) above bars indicate a statistically significant difference (p < 0.05). Groups that share a common letter are not significantly different.

Figure 5. Influence of policosanol on gut microbiota abundance and diversity. (A) Venn diagram of amplicon sequence variants (ASVs) shared among groups. (B) Principal coordinates analysis (PCoA) based on Bray-Curtis distances showing differentiation trends among groups (PERMANOVA, P = 0.0998). (C) Rarefaction curves and alpha diversity indices (Shannon and Simpson). Each group consisted of six replicates (n = 6). The average sequencing depth was 65,974 reads per sample (total reads: 1,187,528). Statistical analysis of differential abundance was performed using ANCOM-BC (FDR q < 0.05).

Figure 6. Gut microbiota composition and differential abundance analysis. (A) Relative abundance of microbial communities at the phylum level. (B) Relative abundance at the genus level (top 10 genera shown). ANCOM-BC analysis identified g__Monoglobus as the only significantly differentially abundant genus (W=85, FDR q < 0.05), though it is not among the top 10 most abundant genera shown here. (C) Partial least squares discriminant analysis (PLS-DA) at the genus level. Each group consisted of six replicates (n = 6). Total sequencing reads: 1,187,528 across 18 samples. Differential abundance analysis using ANCOM-BC (FDR q < 0.05) identified g__Monoglobus as the only significantly differentially abundant genus (W=85).

Figure 7. Classification and variation of metabolites. (A) Proportion of metabolites in each chemical class. (B) Principal component analysis (PCA) scores in positive and negative ion modes. (C) Orthogonal partial least squares discriminant analysis (OPLS-DA) scores for each comparative group. Each group consisted of six replicates (n = 6).

Figure 8. Inter-group differences in metabolites. (A) Volcano plots of differential metabolites. (B) Differential metabolites among three groups. (C) Scatter plot of KEGG pathway enrichment analysis. Each group consisted of six replicates (n = 6).

Figure 9. Correlation networks among growth performance, gut microbiota, and metabolites. (A) Correlation between phenotypic markers (Body Weight, Serum CA, Cortisol, MDA, SOD, GSH-Px) and gut microbiota. Total correlations tested: 180; FDR-significant correlations shown: 19. (B) Correlation between phenotypic markers and differential metabolites. Total correlations tested: 180; FDR-significant correlations shown: 128. (C) Correlation between gut microbiota and metabolites. Total correlations tested: 900; FDR-significant correlations shown: 104. Each group consisted of six replicates (n = 6). Significant correlations in the heatmaps are denoted by asterisks (*, FDR < 0.05; **, FDR < 0.01; ***, FDR < 0.001). Only correlations with FDR-corrected q-value < 0.05 are displayed. Red and blue squares represent positive and negative correlations, respectively, with color intensity corresponding to the absolute value of the correlation coefficient.

**Figure 1**


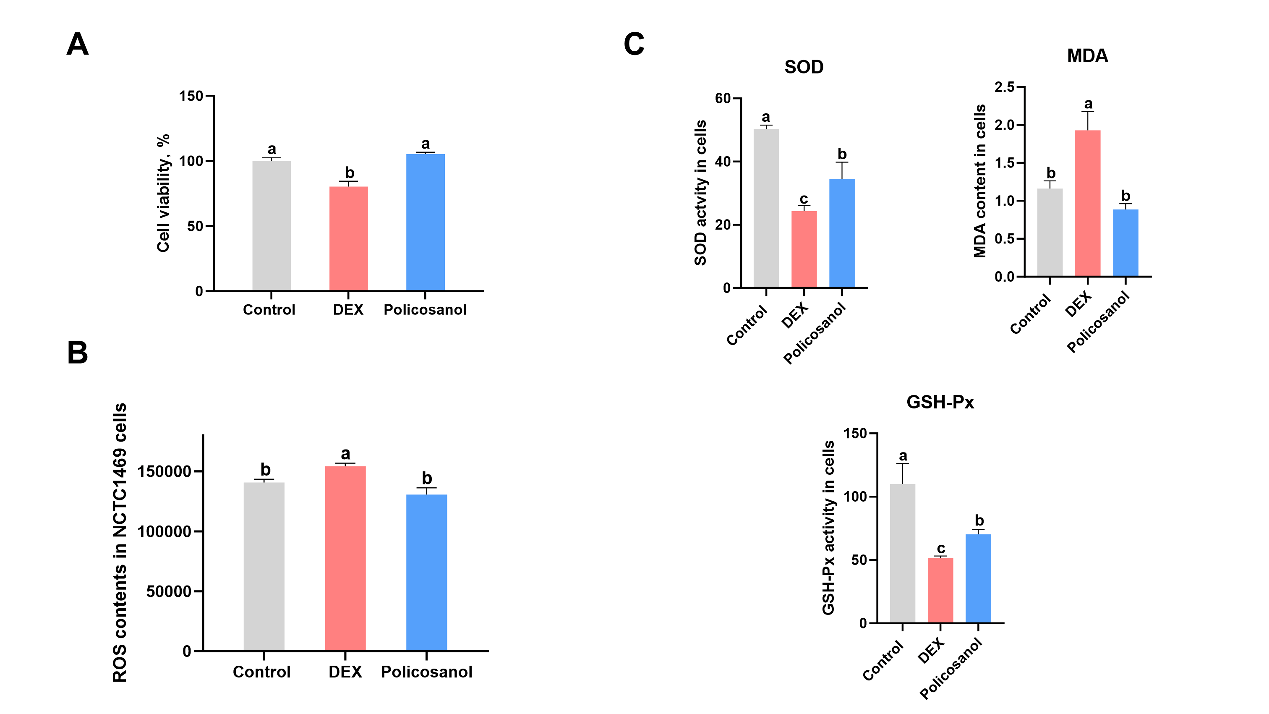


**Figure 2**


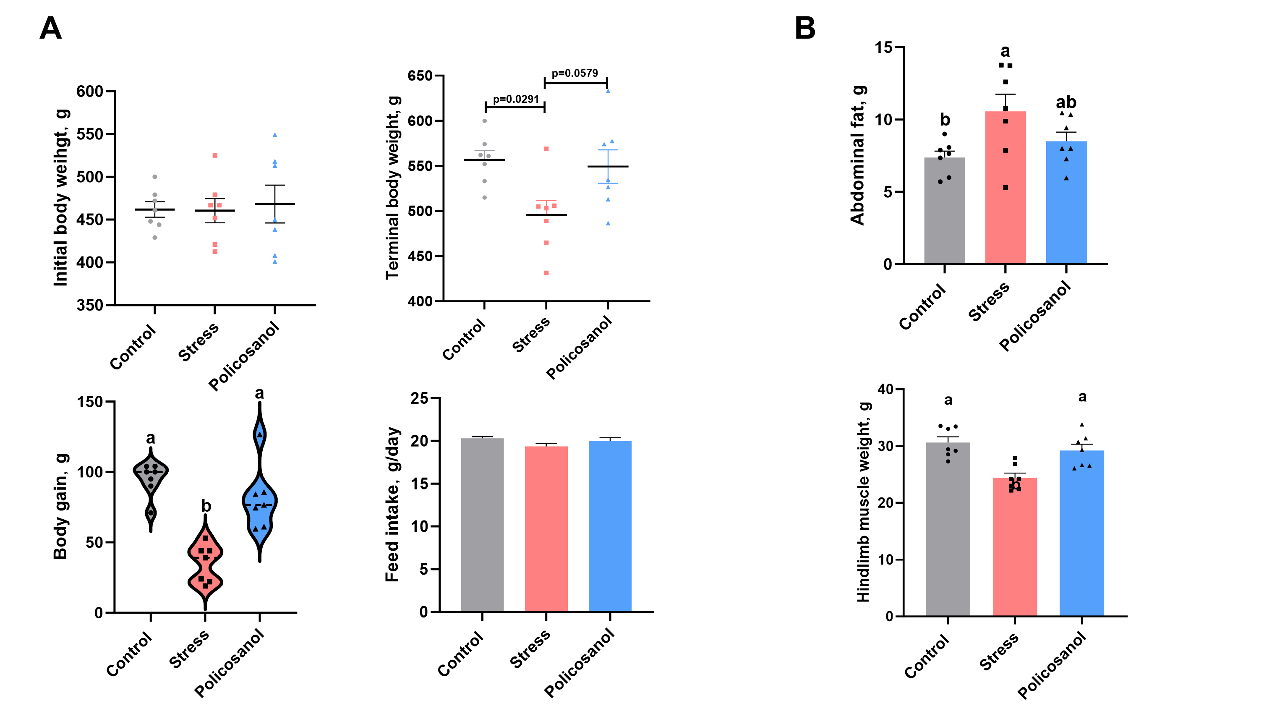


**Figure 3**


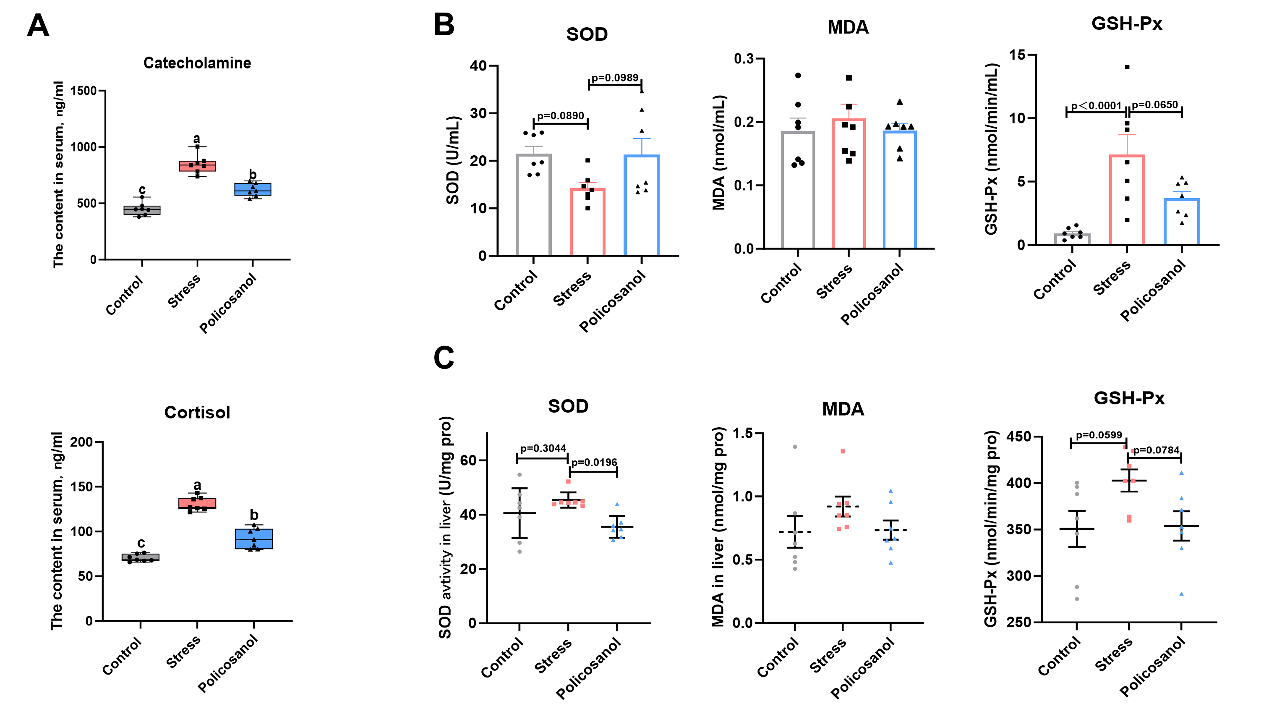


**Figure 4**


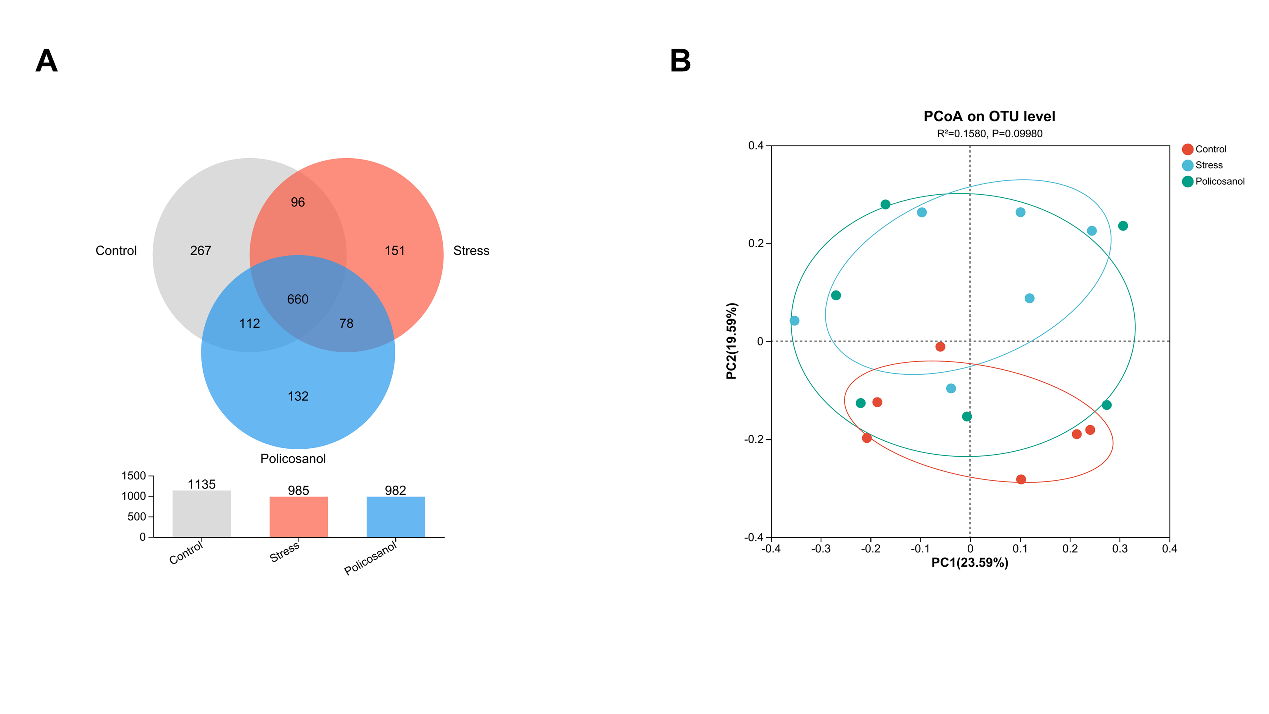


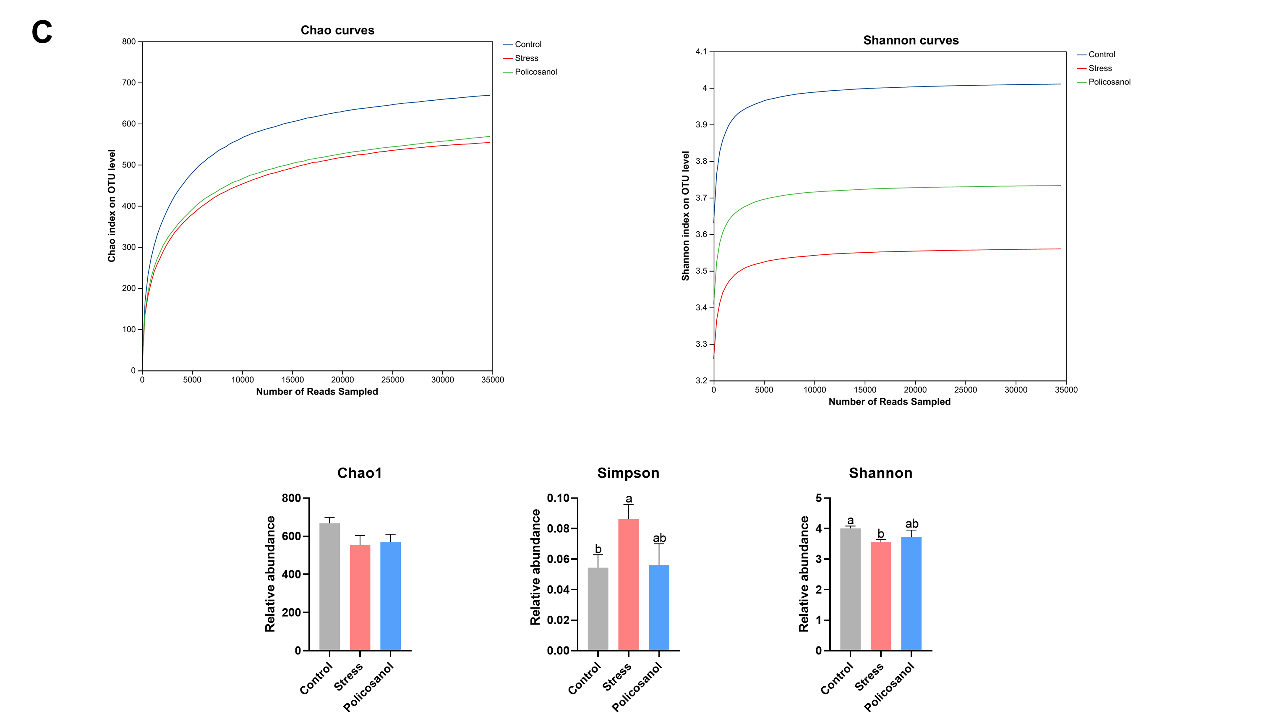


**Figure 5**


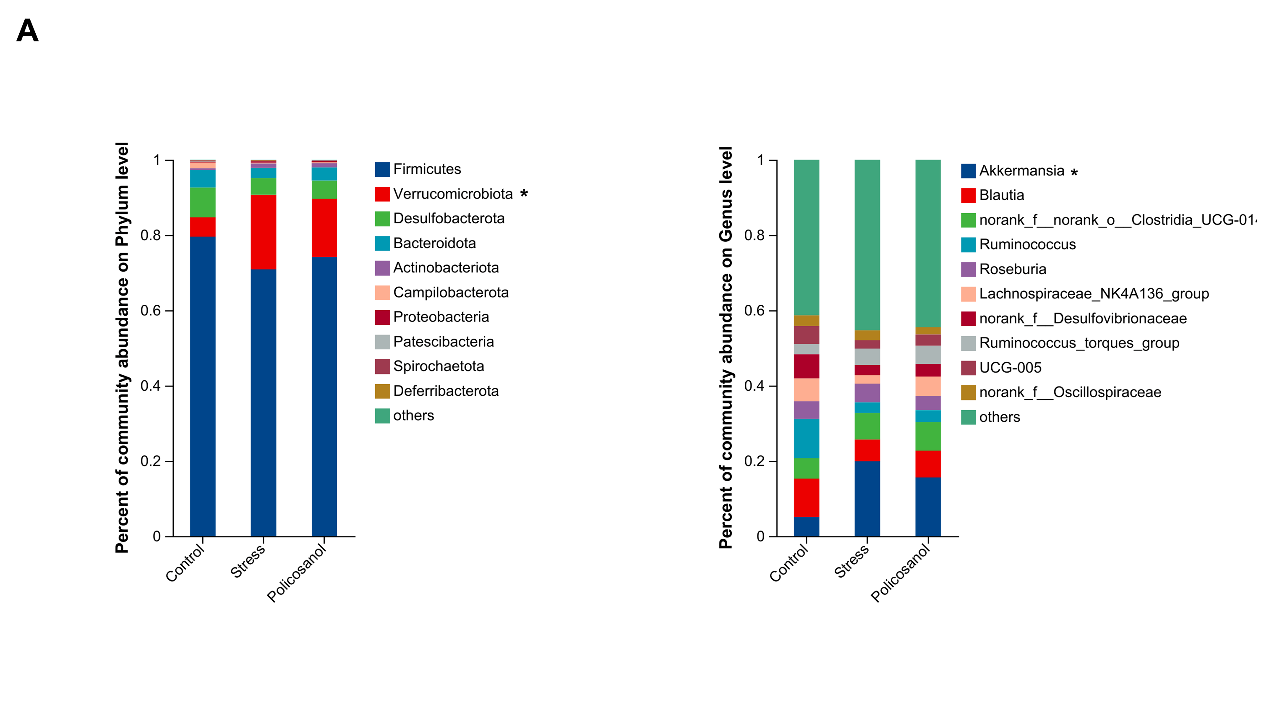


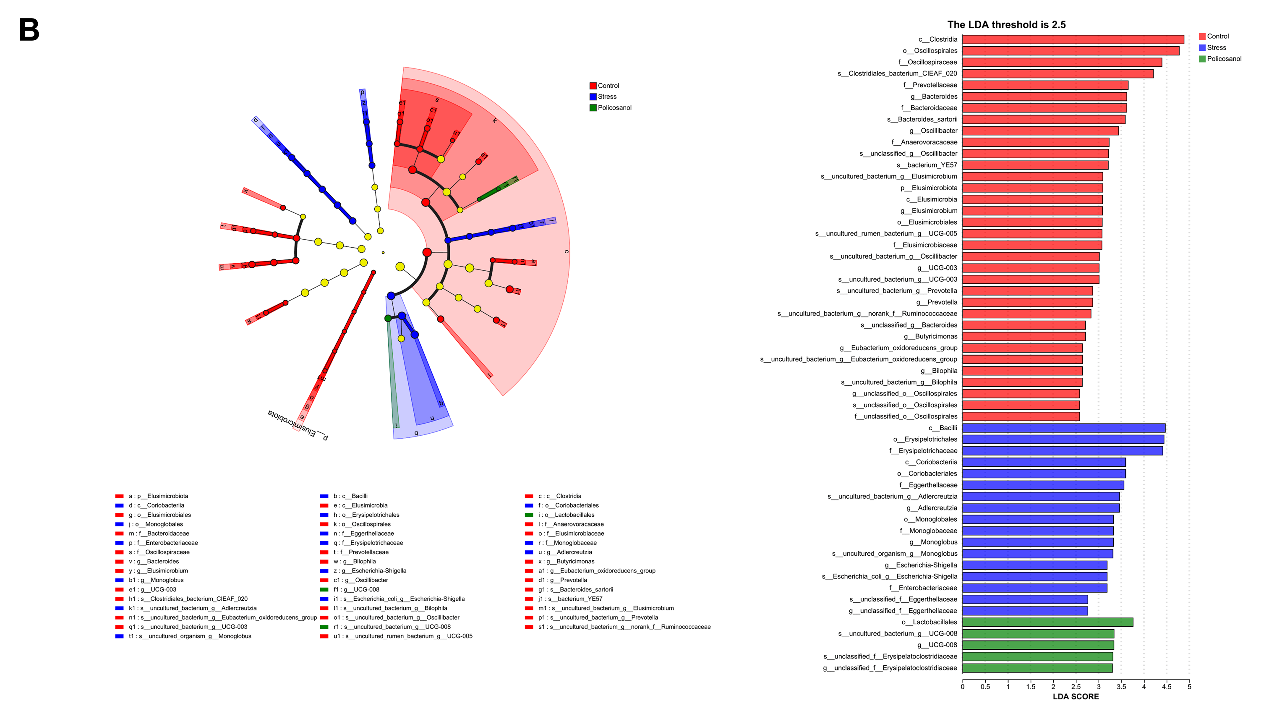


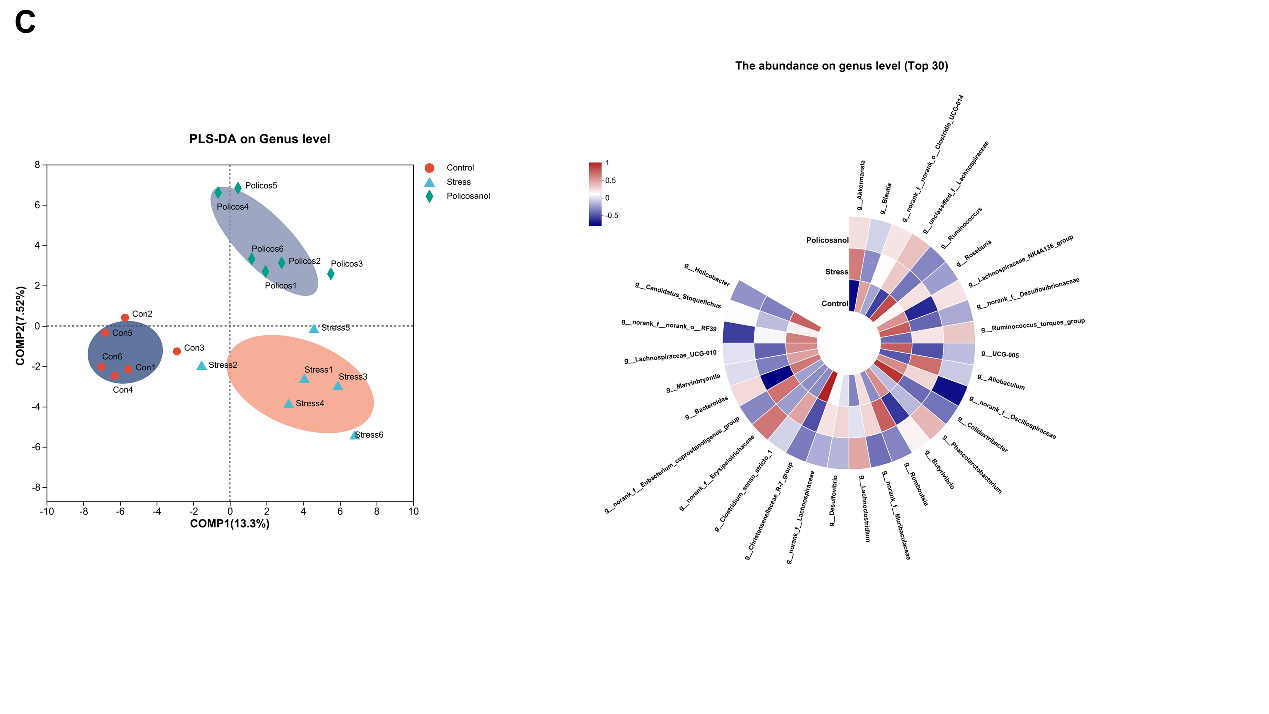


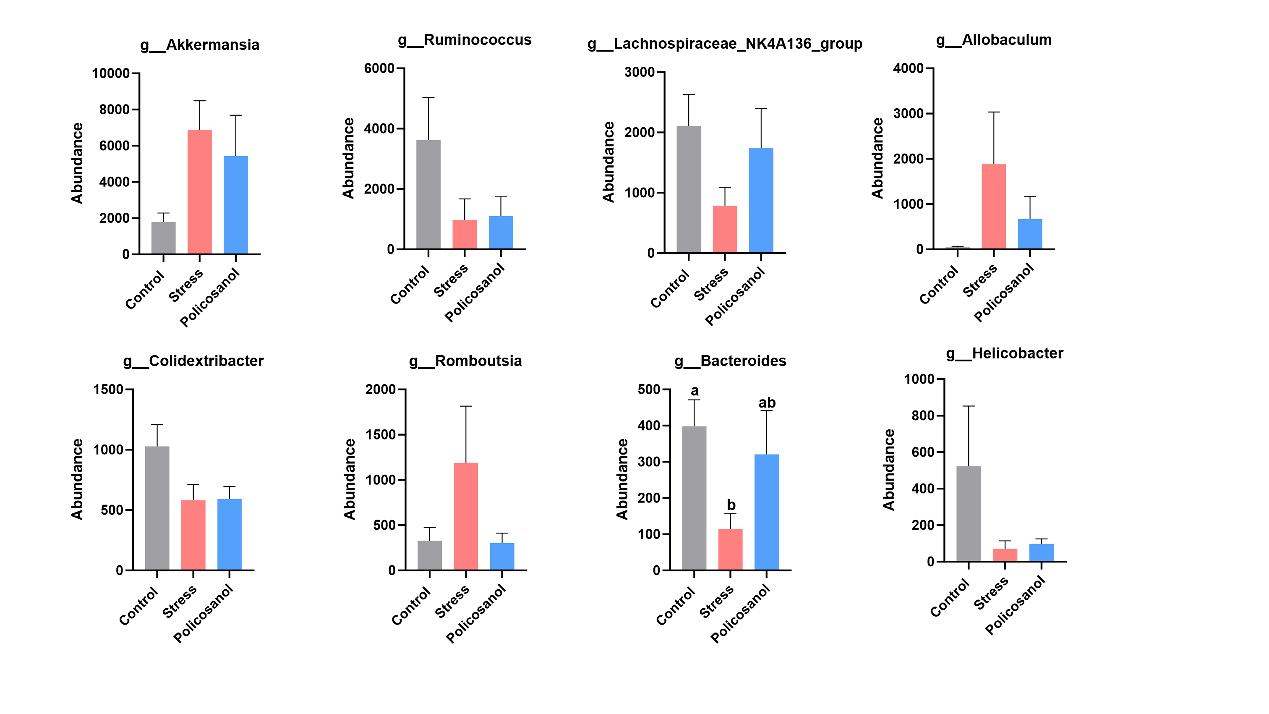


**Figure 6**


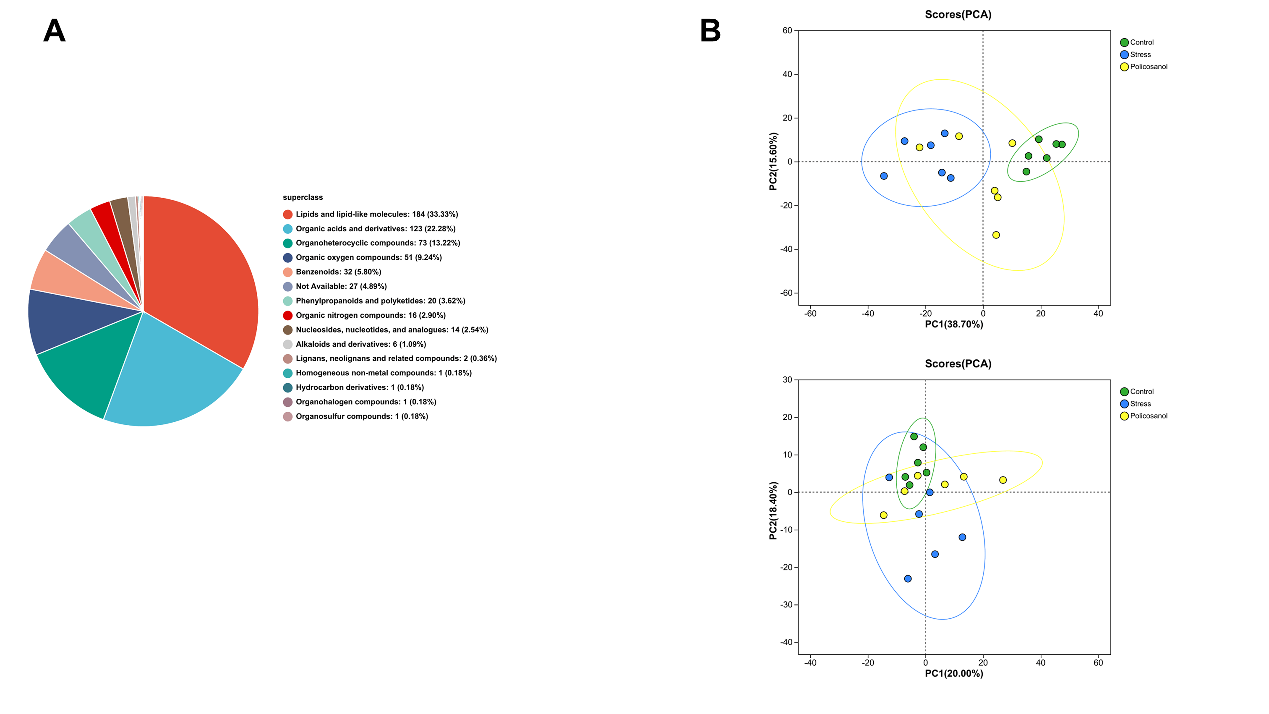


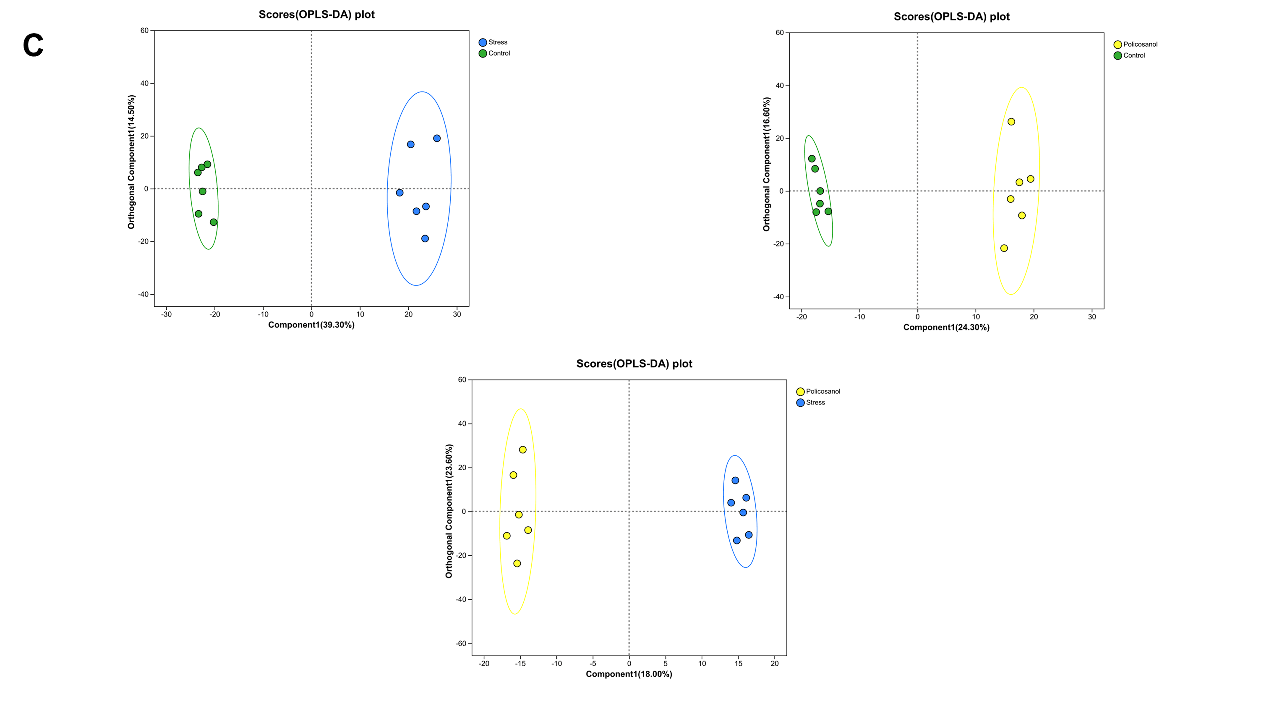


**Figure 7**


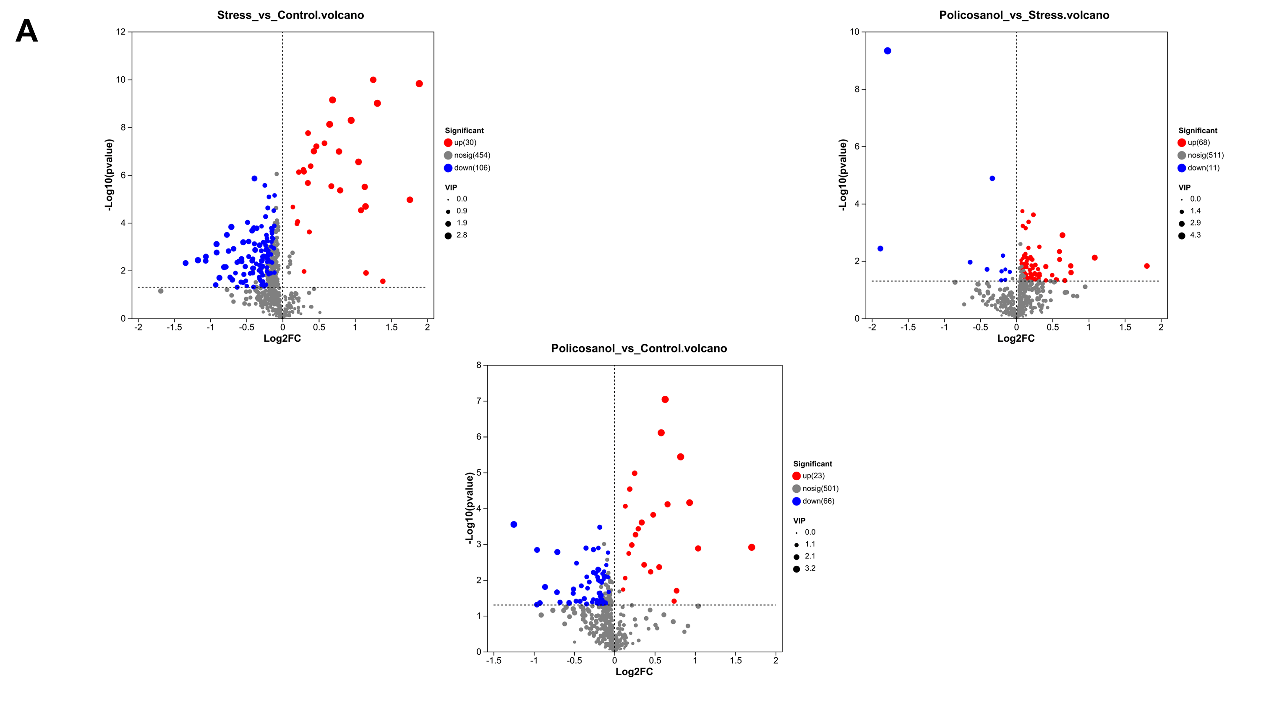


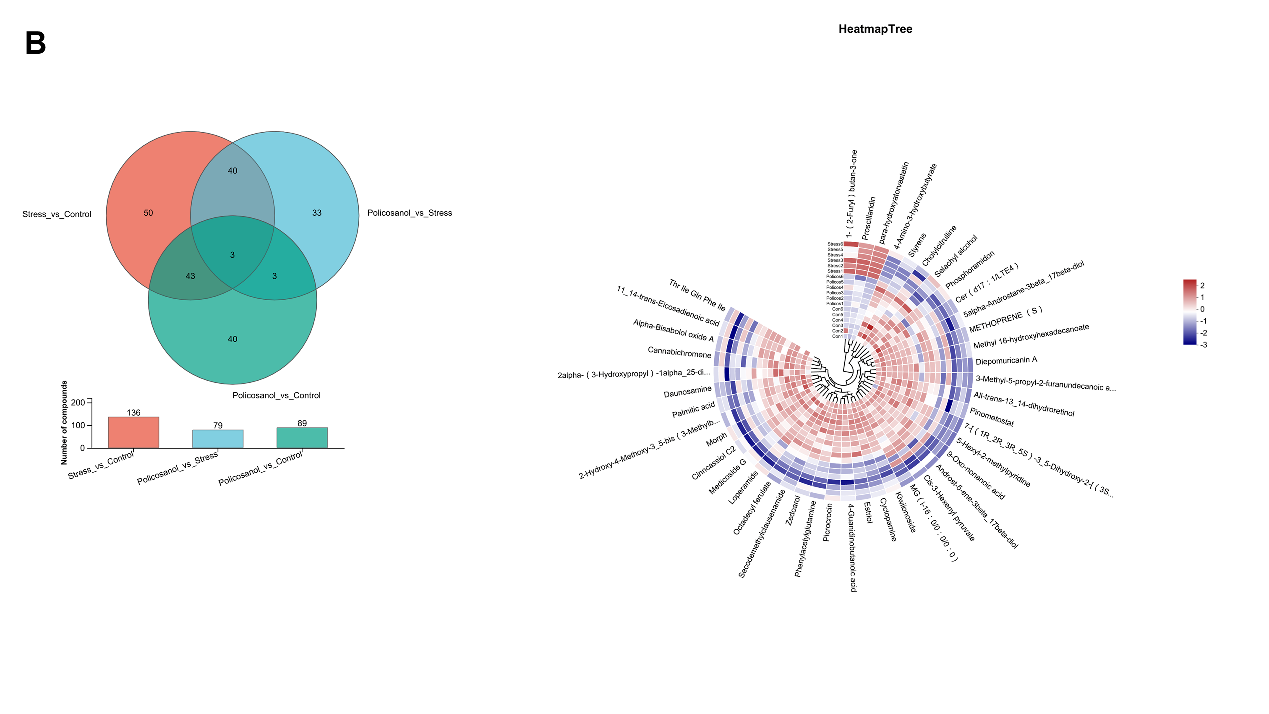


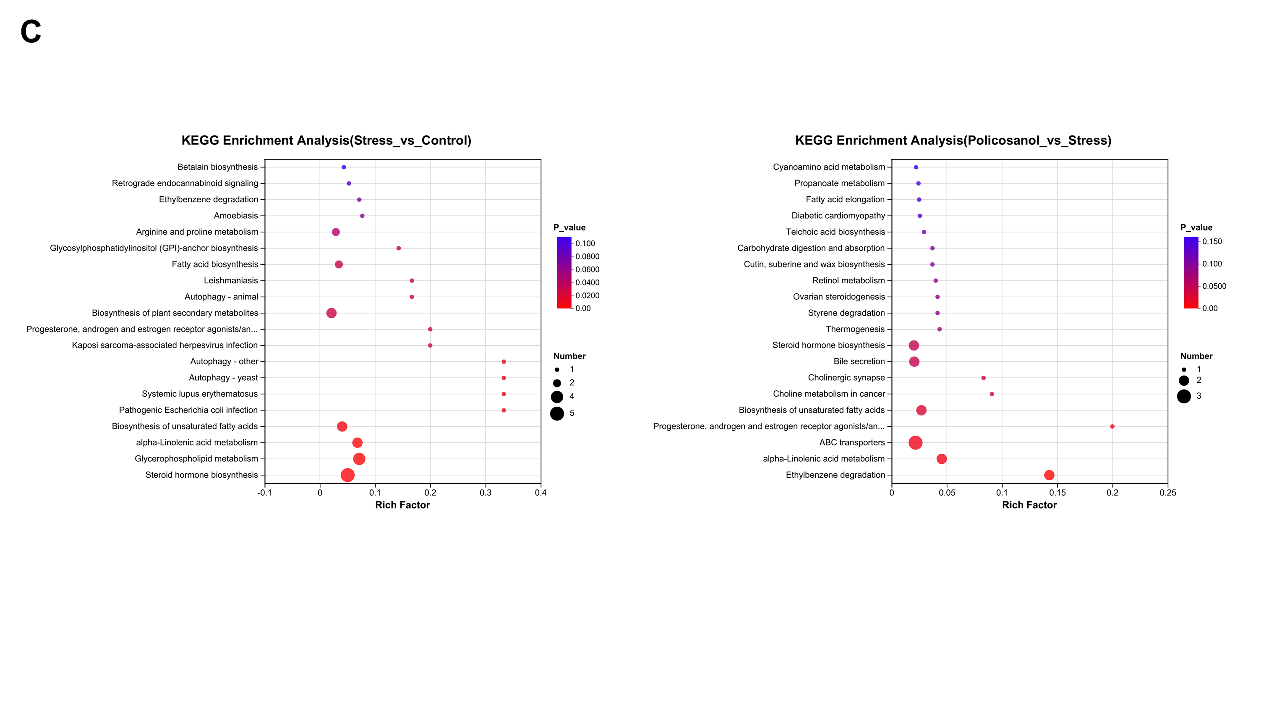


**Figure 8**


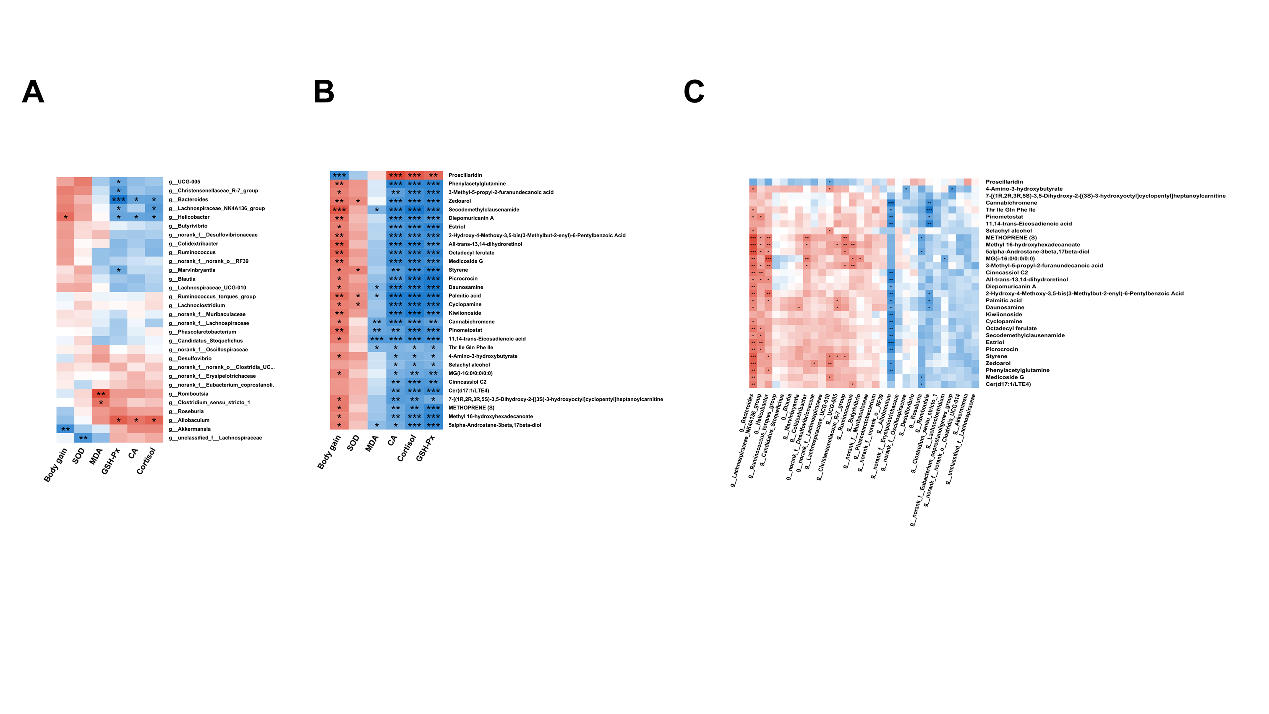

Supplement: Supplementary file 1 [file Data_Sheet_1.zip › Data sheet/figure20251111/figure legend.docx]
