# Supplementary material for: Policosanol alleviates chronic stress-induced growth impairment via gut microbiota-metabolite interactions: insights from 16S rRNA sequencing and LC-MS metabolomics
Source: Front Nutr. 2026 Jan 5;12:1685003. doi: 10.3389/fnut.2025.1685003 (PMC12812650; doi:10.3389/fnut.2025.1685003)

Supplementary Material

**1 Effects of DEX and policosanol dosage**

**1.1. Effect of different concentrations of DEX or Policosanol on the viability of NCTC1469 cells (Supplementary Figure 1)**

NCTC1469 cells were treated with varying concentrations of dexamethasone (DEX) for the indicated time period. Cell viability was assessed using the CCK-8 assay. Data are presented as mean ± SEM.

# Supplementary Figure 2. Schematic timeline of the in vivo experimental design.

# The diagram illustrates the 1-week acclimatization period, the 4-week duration of the daily capture and gavage procedure (applied to the stress and policosanol groups between 8:00-9:00 a.m.), and the terminal sample collection point.
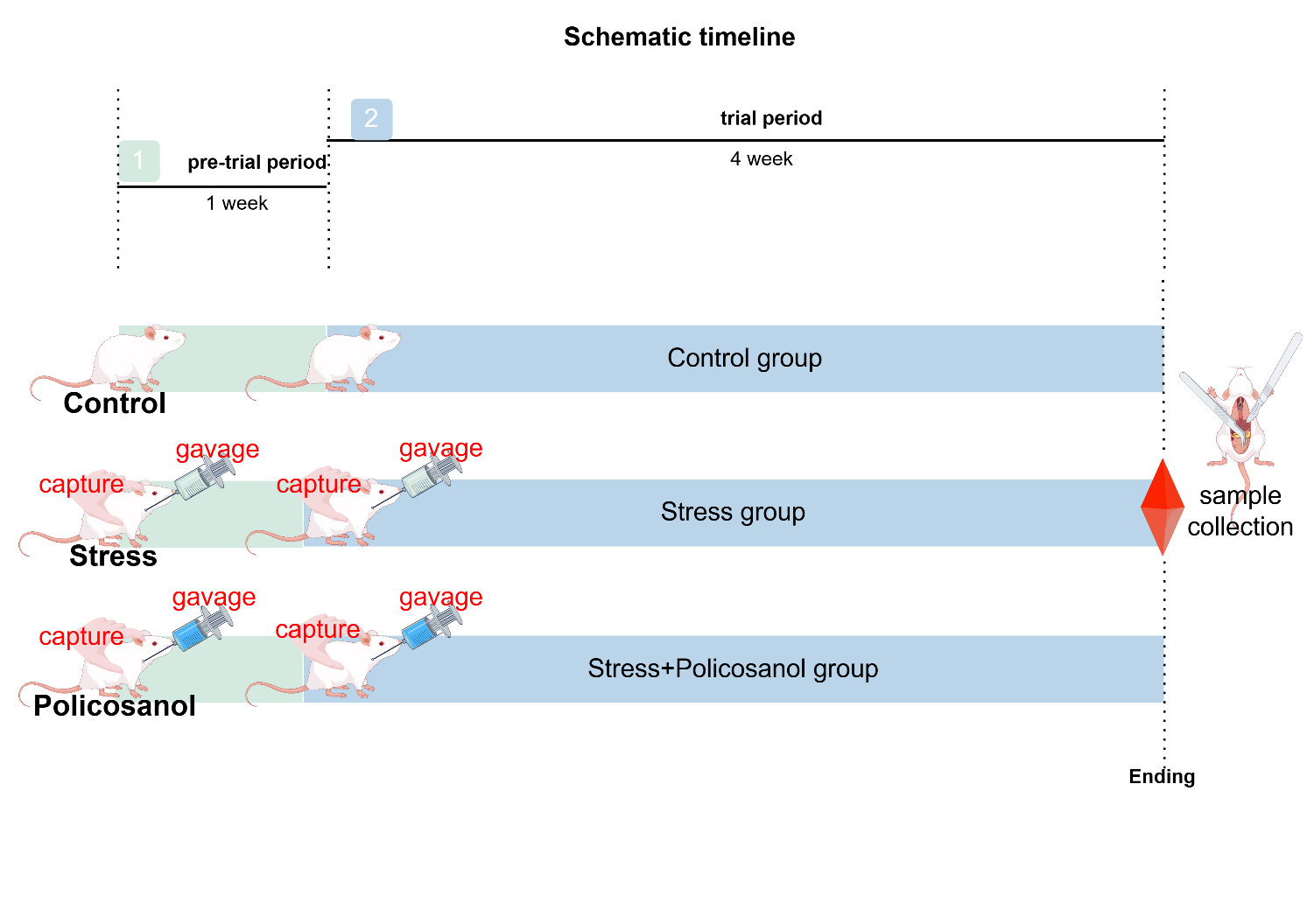


# Supplementary Figure 3. Linear discriminant analysis effect size (LEfSe) analysis identifying differentially abundant taxa.


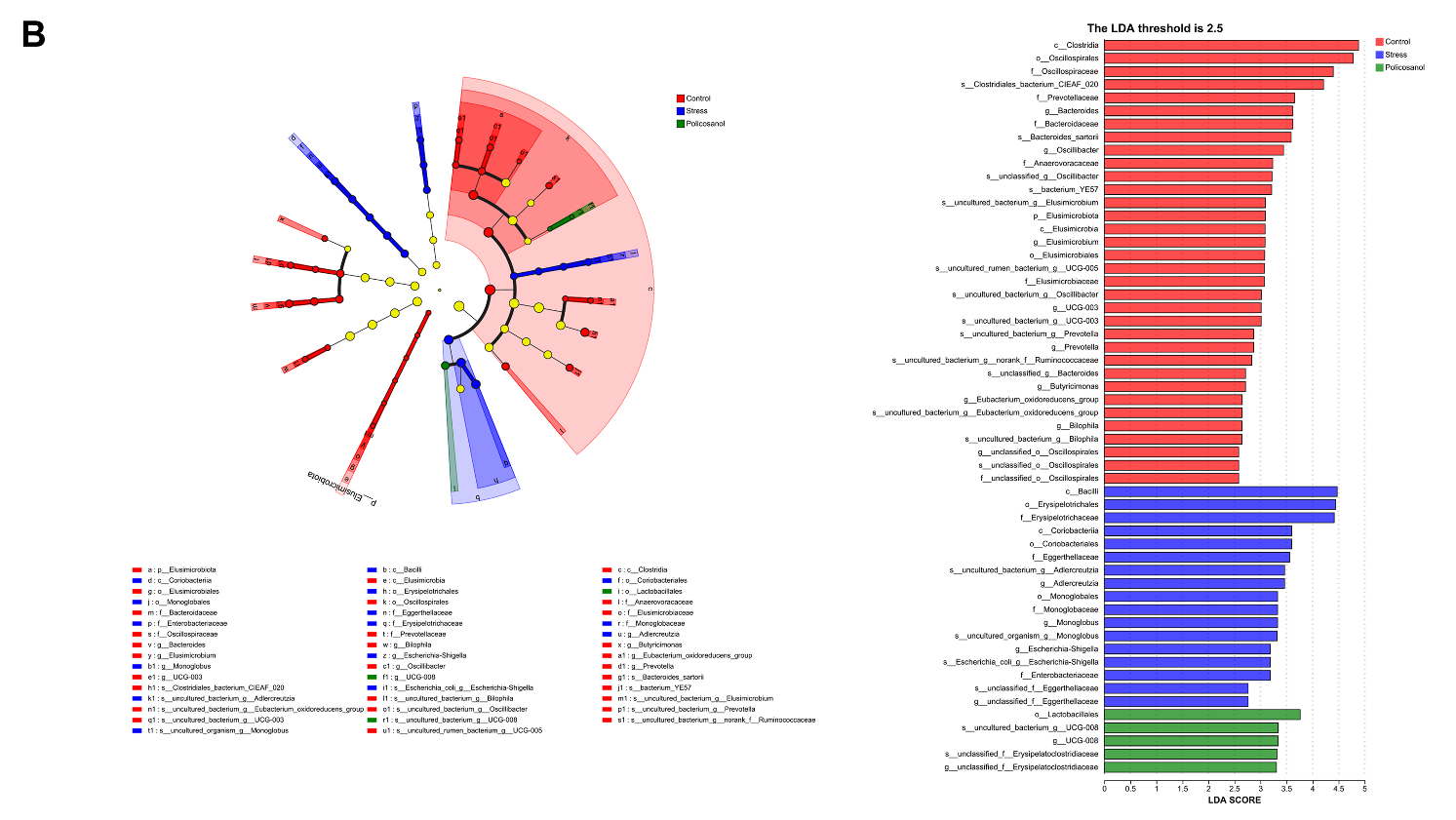


# Supplementary Figure 4. Volcano plots of differential metabolites with q-values <0.05. According to the confidence levels defined by the Metabolomics Standards Initiative (MSI), the identified metabolites in this study were primarily at Level 2 (putative annotation).


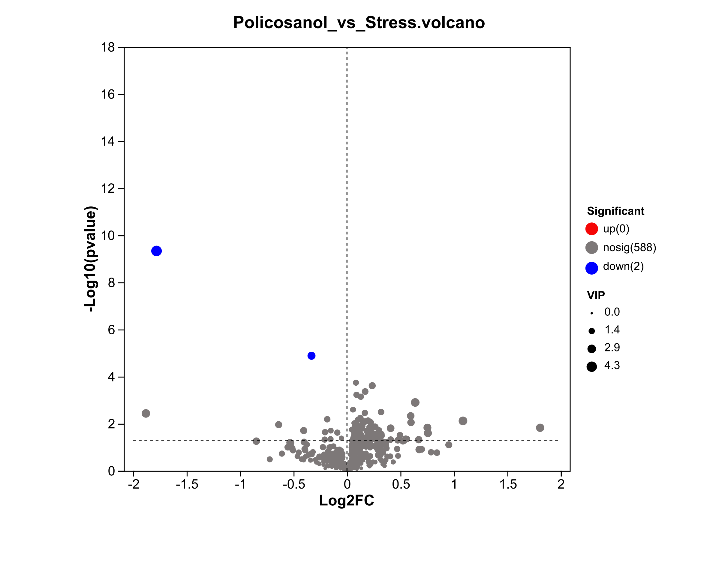

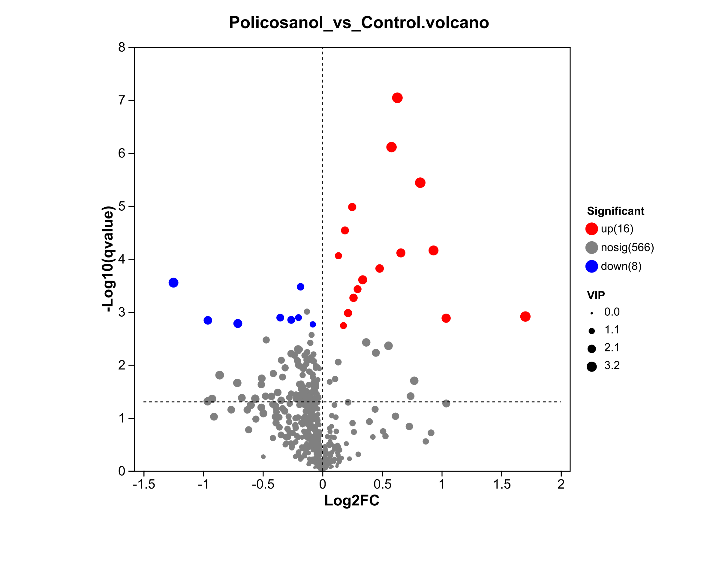


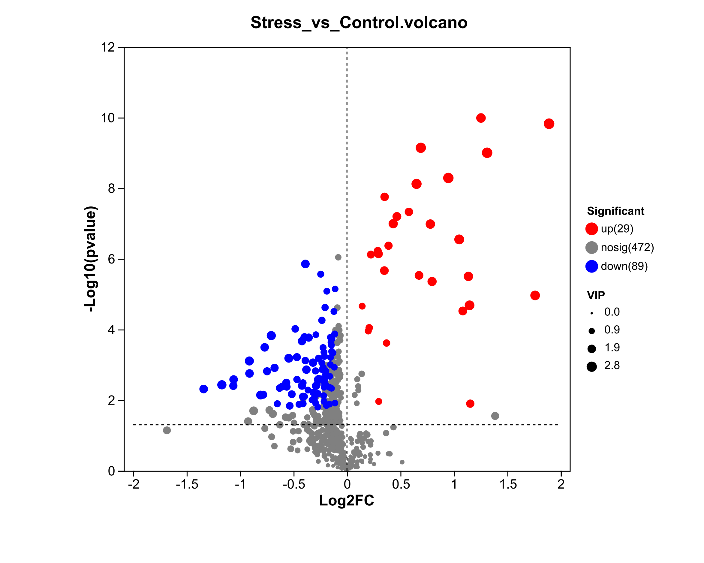

Supplement: Supplementary file 1 [file Data_Sheet_1.zip › Data sheet/Supplementary materials.docx]
