# Supplementary figures and images for: Policosanol alleviates chronic stress-induced growth impairment via gut microbiota-metabolite interactions: insights from 16S rRNA sequencing and LC-MS metabolomics
Source: Front Nutr. 2026 Jan 5;12:1685003. doi: 10.3389/fnut.2025.1685003 (PMC12812650; doi:10.3389/fnut.2025.1685003)

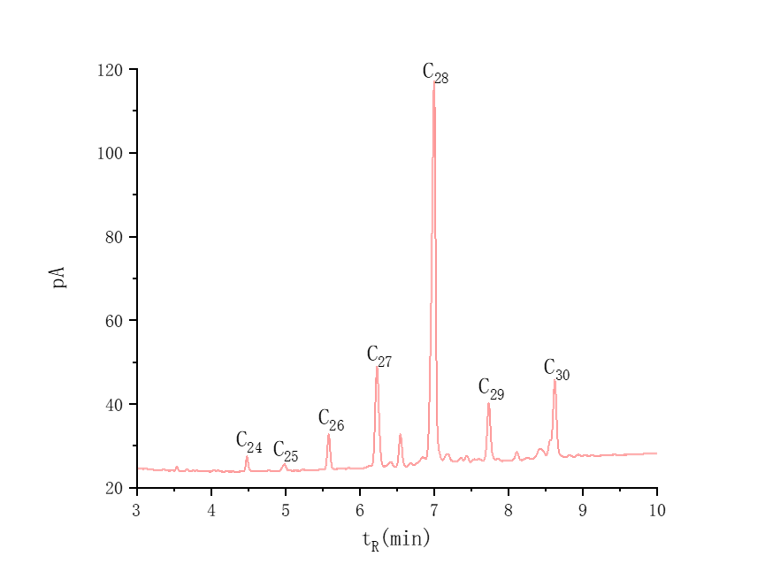

Supplement: Supplementary file 1 [file Data_Sheet_1.zip › Data sheet/figure20251111/Fig1.tif]

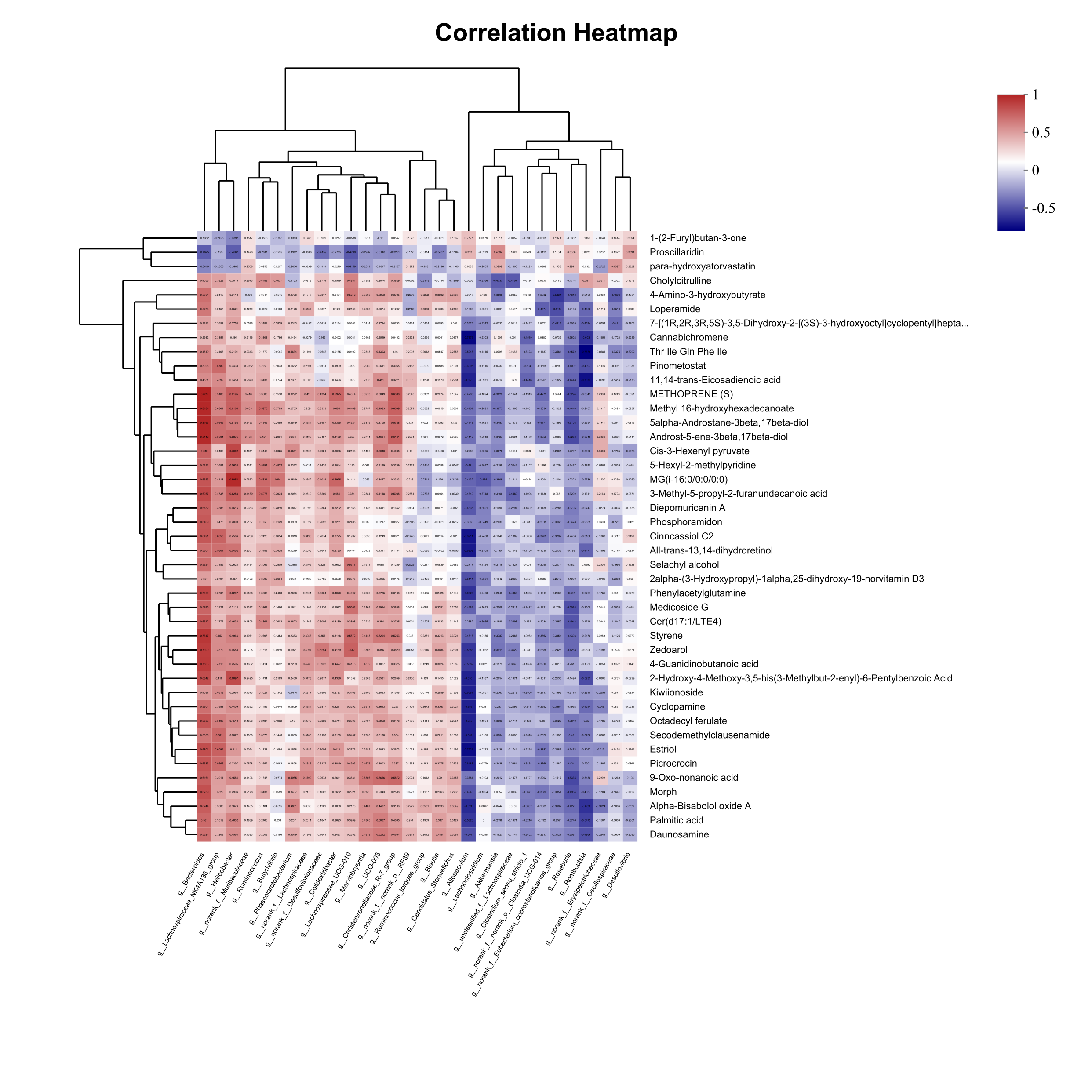

Supplement: Supplementary file 1 [file Data_Sheet_1.zip › Data sheet/S5-Spearman correlation coefficients.tiff]
